# Modeling of Human Prokineticin Receptors: Interactions with Novel Small-Molecule Binders and Potential Off-Target Drugs

**DOI:** 10.1371/journal.pone.0027990

**Published:** 2011-11-21

**Authors:** Anat Levit, Talia Yarnitzky, Ayana Wiener, Rina Meidan, Masha Y. Niv

**Affiliations:** 1 Institute of Biochemistry, Food Science and Nutrition, Faculty of Agriculture, Food and Environment, The Hebrew University of Jerusalem, Rehovot, Israel; 2 Department of Animal Sciences, Faculty of Agriculture, Food and Environment, The Hebrew University of Jerusalem, Rehovot, Israel; 3 The Fritz Haber Center for Molecular Dynamics, The Hebrew University of Jerusalem, Jerusalem, Israel; University of Rome, Italy

## Abstract

**Background and Motivation:**

The Prokineticin receptor (PKR) 1 and 2 subtypes are novel members of family A GPCRs, which exhibit an unusually high degree of sequence similarity. Prokineticins (PKs), their cognate ligands, are small secreted proteins of ∼80 amino acids; however, non-peptidic low-molecular weight antagonists have also been identified. PKs and their receptors play important roles under various physiological conditions such as maintaining circadian rhythm and pain perception, as well as regulating angiogenesis and modulating immunity. Identifying binding sites for known antagonists and for additional potential binders will facilitate studying and regulating these novel receptors. Blocking PKRs may serve as a therapeutic tool for various diseases, including acute pain, inflammation and cancer.

**Methods and Results:**

Ligand-based pharmacophore models were derived from known antagonists, and virtual screening performed on the DrugBank dataset identified potential human PKR (hPKR) ligands with novel scaffolds. Interestingly, these included several HIV protease inhibitors for which endothelial cell dysfunction is a documented side effect. Our results suggest that the side effects might be due to inhibition of the PKR signaling pathway. Docking of known binders to a 3D homology model of hPKR1 is in agreement with the well-established canonical TM-bundle binding site of family A GPCRs. Furthermore, the docking results highlight residues that may form specific contacts with the ligands. These contacts provide structural explanation for the importance of several chemical features that were obtained from the structure-activity analysis of known binders. With the exception of a single loop residue that might be perused in the future for obtaining subtype-specific regulation, the results suggest an identical TM-bundle binding site for hPKR1 and hPKR2. In addition, analysis of the intracellular regions highlights variable regions that may provide subtype specificity.

## Introduction

### Prokineticins and their receptors

Mammalian prokineticins 1 and 2 (PK1 and PK2) are two secreted proteins of about 80–90 residues in length, which belong to the AVIT protein family [1], [2], [3]. Their structure includes 10 conserved cysteine residues that create five disulphide-bridged motifs (colipase fold) and an identical (AVIT) motif in the N-terminus.

PKs are expressed in a wide range of peripheral tissues, including the nervous, immune, and cardiovascular systems, as well as in the steroidogenic glands, gastrointestinal tract, and bone marrow [3], [4], [5], [6].

**Figure 1:**
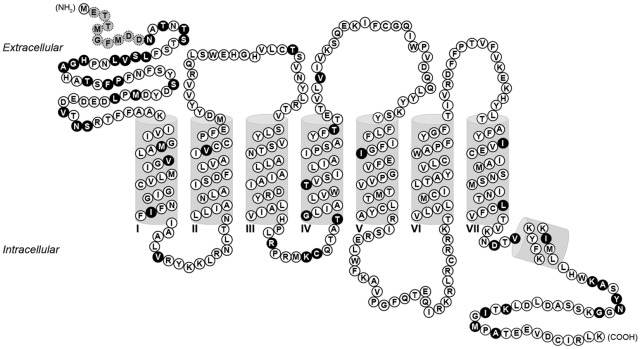
Snake plot of hPKR1. The secondary structure is according to hPKR1 protein annotation in the UniProtKB database (entry Q8TCW9). Positions in the hPKR1 sequence differing from hPKR2 (entry Q8NFJ6) are shaded black. Conserved positions between the two subtypes are shaded white. A nine-residue hPKR1-unique insert in the N terminus is shaded gray with dashed lines. The seven transmembrane domains are denoted by roman numerals. Extracellular and intracellular sides of the membrane are labeled, as well as the N terminus (NH_2_) and C terminus (COOH) ends of the protein.

PKs serve as the cognate ligands for two highly similar G-protein-coupled receptors (GPCRs) termed PKs receptor subtypes 1 and 2 (hPKR1 and hPKR2 in humans) [5], [7], [8]. These receptors are characterized by seven membrane-spanning α-helical segments separated by alternating intracellular and extracellular loop regions. The two subtypes are unique members of family A GPCRs in terms of subtype similarity, sharing 85% sequence identity – a particularly high value among known GPCRs. For example, the sequence identity between the β1 and β2-adrenergic receptor subtypes, which are well established drug targets, is 57%. Most sequence variation between the hPKR subtypes is concentrated in the extracellular N terminal region, which contains a nine-residue insert in hPKR1 compared with hPKR2, as well as in the second intracellular loop (ICL2) and in the C terminal tail (Figure 1).

PKR1 is mainly expressed in peripheral tissues, such as the endocrine organs and reproductive system, the gastrointestinal tract, lungs, and the circulatory system [8], [9], whereas PKR2, which is also expressed in peripheral endocrine organs [8], is the main subtype in the central nervous system. Interestingly, PKR1 is expressed in endothelial cells of large vessels while PKR2 is strongly expressed in fenestrated endothelial cells of the heart and corpus luteum [10], [11]. Expression analysis of PKRs in heterogeneous systems revealed that they bind and are activated by nanomolar concentrations of both recombinant PKs, though PK2 was shown to have a slightly higher affinity for both receptors than was PK1 [12]. Hence, in different tissues, specific signaling outcomes following receptor activation may be mediated by different ligand-receptor combinations, in accordance with the expression profile of both ligands and receptors in that tissue [13]. Activation of PKRs leads to diverse signaling outcomes, including mobilization of calcium, stimulation of phosphoinositide turnover, and activation of the p44/p42 MAPK cascade in overexpressed cells, as well as in endothelial cells naturally expressing PKRs [5], [7], [8], [14], [15] leading to the divergent functions of PKs. Differential signaling capabilities of the PKRs is achieved by coupling to several different G proteins, as previously demonstrated [11].

The PKR system is involved in different pathological conditions such as heart failure, abdominal aortic aneurysm, colorectal cancer, neuroblastoma, polycystic ovary syndrome, and Kallman syndrome [16]. While Kallman syndrome is clearly linked to mutations in the PKR2 gene, it is not currently established whether the other diverse biological functions and pathological conditions are the result of a delicate balance of both PKR subtypes or depend solely on one of them.

Recently, small-molecule, non-peptidic PKR antagonists have been identified through a high-throughput screening procedure [17], [18], [19], [20]. These guanidine triazinedione-based compounds competitively inhibit calcium mobilization following PKR activation by PKs in transfected cells, in the nanomolar range [17]. However, no selectivity for one of the subtypes has been observed [17].

A better understanding of the PK system can generate pharmacological tools that will affect diverse areas such as development, immune response, and endocrine function. Therefore, the molecular details underlying PK receptor interactions, both with their cognate ligands and small-molecule modulators, and with downstream signaling partners, as well as the molecular basis of differential signaling, are of great fundamental and applied interest.

Structural information has been instrumental in delineating interactions and the rational development of specific inhibitors [21]. However, for many years only the X-ray structure of bovine Rhodopsin has been available [22] as the sole representative structure of the large superfamily of seven-transmembrane (7TM) domain GPCRs.

In recent years crystallographic data on GPCRs has significantly grown and now includes, for example, structures of the β1 and β2-adrenergic receptors, in both active and inactive states, the agonist- and antagonist-bound A_2A_ adenosine receptor, and the CXCR4 chemokine receptor bound to small-molecule and peptide antagonists. The new structures were reviewed in [23], [24] and ligand-receptor interactions were summarized in [25]. Nevertheless, the vast number of GPCR family members still requires using computational 3D models of GPCRs for studying these receptors and for drug discovery. Different strategies for GPCR homology modeling have been developed in recent years (reviewed in [26]), and these models have been successfully used for virtual ligand screening (VLS) procedures, to identify novel GPCR binders [21].

Successful *in-silico* screening approaches, applied to GPCR drug discovery, include both structure-based and ligand-based techniques and their combinations. Molecular ligand docking is the most widely used computational structure-based approach, employed to predict whether small-molecule ligands from a compound library will bind to the target's binding site. When a ligand-receptor complex is available, either from an X-ray structure or an experimentally verified model, a structure-based pharmacophore model describing the possible interaction points between the ligand and the receptor can be generated using different algorithms and later used for screening compound libraries [27]. In ligand-based VLS procedures, the pharmacophore is generated via superposition of 3D structures of several known active ligands, followed by extracting the common chemical features responsible for their biological activity. This approach is often used when no reliable structure of the target is available [28].

In this study, we analyzed known active small-molecule antagonists of hPKRs vs. inactive compounds to derive ligand-based pharmacophore models. The resulting highly selective pharmacophore model was used in a VLS procedure to identify potential hPKR binders from the DrugBank database. The interactions of both known and predicted binders with the modeled 3D structure of the receptor were analyzed and compared with available data on other GPCR-ligand complexes. This supports the feasibility of binding in the TM-bundle and provides testable hypotheses regarding interacting residues. The potential cross-reactivity of the predicted binders with the hPKRs was discussed in light of prospective 'off-target' effects. The challenges and possible venues for identifying subtype-specific binders are addressed in the discussion section.

## Materials and Methods

### Homology Modeling and Refinement

All-atom homology models of human PKR1 and PKR2 were generated using the I-TASSER server [29], which employs a fragment-based method. Here a hierarchical approach to protein structure modeling is used in which fragments are excised from multiple template structures and reassembled, based on threading alignments. Sequence alignment of modeled receptor subtypes and the structural templates were generated by the TCoffee server [30]; this information is available in the Supporting Information as figure S1. A total of 5 models per receptor subtype were obtained. The model with the highest C-score (a confidence score calculated by I-Tasser) for each receptor subtype, was exported to Discovery Studio 2.5 (DS2.5; Accelrys, Inc.) for further refinement. In DS2.5, the model quality was assessed using the protein report tool, and the models were further refined by energy minimization using the CHARMM force field [31]. The models were then subjected to side-chain refinement using the SCWRL4 program [32], and to an additional round of energy minimization using the Smart Minimizer algorithm, as implemented in DS2.5. The resulting models were visually inspected to ensure that the side chains of the most conserved residues in each helix are aligned to the templates. An example of these structural alignments appears in figure S2.

For validation purposes, we also generated homology models of the turkey β1 adrenergic receptor (β1adr) and the human β2 adrenergic receptor (β2adr). The β1adr homology model is based on 4 different β2adr crystal structures (PDB codes – 3SN6, 2RH1, 3NY8, and 3d4S); the β2adr model is based on the crystal structures of β1adr (2VT4, 2YCW), the Dopamine D3 receptor (3PBL), and the histamine H1 receptor (3RZE). The models were subjected to the same refinement procedure as previously described, namely, deletion of loops, energy minimization, and side chain refinement, followed by an additional step of energy minimization. Sometimes the side chain rotamers were manually adjusted, following the aforementioned refinement procedure.

Throughout this article, receptor residues are referred to by their one-letter code, followed by their full sequence number in hPKR1. TM residues also have a superscript numbering system according to Ballesteros-Weinstein numbering [33]; the most conserved residue in a given TM is assigned the index X.50, where X is the TM number, and the remaining residues are numbered relative to this position.

### Identification of a 7TM-bundle binding site

The location of a potential small-molecule-TM binding cavity was identified based on (1) identification of receptor cavities using the "eraser" and "flood-filling" algorithms [34], as implemented in DS2.5 and (2) use of two energy-based methods that locate energetically favorable binding sites – Q-SiteFinder [35], an algorithm that uses the interaction energy between the protein and a simple Van der Waals probe to locate energetically favorable binding sites, and SiteHound [36], which uses a carbon probe to similarly identify regions of the protein characterized by favorable interactions. A common site that encompasses the results from the latter two methods was determined as the TM-bundle binding site for small molecules.

### SAR Analysis

A dataset of 107 small-molecule hPKR antagonists was assembled from the literature [18], [19]. All ligands were built using DS2.5. pKa values were calculated for each ionazable moiety on each ligand, to determine whether the ligand would be charged and which atom would be protonated at a biological pH of 7.5. All ligands were then subjected to the "Prepare Ligands" protocol, to generate tautomers and enantiomers, and to set standard formal charges.

For the SAR study, the dataset was divided into two parts: (1) active molecules, with IC_50_ values below 0.05 µM, and (2) inactive molecules, with IC_50_ values above 1 µM. IC_50_ values were measured in the calcium mobilization assay [18], [19]. When possible, the molecules were divided into pairs of active and inactive molecules that differ in only one chemical group, and all possible pharmacophore features were computed using the "Feature mapping" protocol (DS 2.5). These pairs were then compared to determine those pharmacophore features' importance for biological activity.

### Ligand-Based Pharmacophore Models

The HipHop algorithm [37], implemented in DS2.5, was used for constructing ligand-based pharmacophore models. This algorithm derives common features of pharmacophore models using information from a set of active compounds. The two most active hPKR antagonists (the lowest IC_50_ values in the Janssen patent [19], [20]) were selected as ‘reference compounds’ from the data set described above, and an additional antagonist molecule with a different scaffold was added from a dataset recently published [38], and were used to generate the models (figure S3). Ten models in total were generated, presenting different combinations of chemical features. These models were first evaluated by their ability to successfully recapture all known active hPKR antagonists. An enrichment study was performed to evaluate the pharmacophore models. The dataset contains 56 active PKR antagonists seeded in a random library of 5909 decoys retrieved from the ZINC database [39]. The decoys were selected so that they will have general and chemical properties similar to the known hPKR antagonists (by filtering the ZINC database according to the average molecular properties of known hPKR antagonists ± 4 Standard Deviation range). In this way, enrichment is not simply achieved by separating trivial features (such as mass, overall charge, etc.). These properties included AlogP (a log of the calculated octanol-water partition coefficient, which measures the extent of a substance hydrophilicity or hydrophobicity), molecular weight, formal charge, the number of hydrogen bond donors and acceptors, and the number of rotatable bonds. All molecules were prepared as previously described, and a conformational set of 50 "best-quality" low-energy conformations was generated for each molecule. All conformers within 20 kcal/mol from the global energy minimum were included in the set. The dataset was screened using the "ligand pharmacophore mapping" protocol (DS2.5), with the minimum interference distance set to 1Å and the maximum omitted features set to 0. All other protocol parameters were maintained at the default settings.

To analyze enrichment results and select the best pharmacophore model for subsequent virtual screening, ROC curves were constructed for each model, where the fraction of identified known binders (true positives, representing sensitivity) was plotted against the fraction of identified library molecules (false positives; 1-specificity). Based on this analysis, the best pharmacophore model was selected for virtual screening purposes.

### Generation of the DrugBank data set and virtual screening

The DrugBank database [40] (release 2.0), which contains ∼4900 drug entries, including 1382 FDA-approved small-molecule drugs, 123 FDA-approved biotech (protein/peptide) drugs, 71 nutraceuticals, and over 3240 experimental drugs, was used for Virtual Screening. The database was filtered, based on the average molecular properties of known hPKR antagonists ± 4SD (standard deviation). These properties included AlogP, molecular weight, the number of hydrogen bond donors and acceptors, the formal charge, and the number of rotatable bonds. The liberal ±4SD interval was chosen because the calculated range of molecular properties of the known antagonists was very narrow. Molecules were retained only if their formal charge was neutral or positive, since the known compounds were positively charged. This resulted in a test set containing 432 molecules. All molecules were prepared as previously described, and a set of 50 "best-quality" low-energy conformations was generated for each molecule; all conformations were within 20 kcal/mol from the global energy minimum.

The data set was screened against the pharmacophore model (chosen from the ROC analysis) using the "ligand pharmacophore mapping" protocol in DS2.5. All protocol settings were maintained at default settings except for minimum interference distance, which was set to 1Å and the maximum omitted features was set to 0. To prioritize the virtual hits, fit values were extracted, to reflect the quality of molecule mapping onto the pharmacophore. Only molecules with fit values above the enrichment ROC curve cutoff that identifies 100% of the known PKR antagonists (FitValue≥2.85746) were retained as virtual hits for further analysis.

The similarity between the virtual hits and known small-molecule PKR antagonists was evaluated by calculating the Tanimoto coefficient distance measure using the 'Find similar molecules by fingerprints' module in DS2.5, which calculates the number of AND bits normalized by the number of OR bits, according to *SA/(SA+SB+SC)*, where *SA* is the number of AND bits (bits present in both the target and the reference), *SB* is the number of bits in the target but not the reference, and *SC* is the number of bits in the reference but not the target.

### Small-Molecule Docking

Molecular docking of the small-molecule hPKR antagonists dataset (active and inactive molecules), as well as of virtual hits, to the hPKR1 homology model, was performed using LigandFit [34] as implemented in DS2.5. LigandFit is a shape complementary-based algorithm that performs flexible ligand-rigid protein docking. In our experiments, the binding site was defined as a 284.8 Å^3^ TM cavity area, surrounded by binding site residues identified using the energy-based methods described above. Default algorithm settings were used for docking. The final ligand poses were selected based on their empirical LigScore docking score [41]. Here we used the (default) Dreiding force field to calculate the VdW interactions.

All docking experiments were conducted on a model without extracellular and intracellular loops. Loop configurations are highly variable among the GPCR crystal structures [42]. Therefore, deleting the loops in order to reduce the uncertainty stemming from inaccurately predicted loops is a common practice in the field [43], [44], [45].

To further validate our protocol, we also performed molecular redocking of the small-molecule partial inverse agonist carazolol and the antagonist cyanopindolol to their original X-ray structures from which loops were deleted, and to loopless homology models of β1adr and β2adr using LigandFit, as previously described. As in the case of docking to the hPKR1 model, this procedure was performed on loopless X-ray structures and models. The binding site was identified from receptor cavities using the "eraser" and "flood-filling" algorithms, as implemented in DS2.5. The highest scoring LigScore poses were selected as the representative solutions. The ligand-receptor poses were compared to the corresponding X-ray complexes by (1) calculating the root mean square deviation (RMSD) of heavy ligand atoms from their respective counterparts in the crystallized ligand after superposition of the docked ligand-receptor complex onto the X-ray structure; (2) calculating the number of correct atomic contacts in the docked ligand-receptor complex compared with the X-ray complex, where an atomic contact is defined as a pair of heavy ligand and protein atoms located at a distance of less than 4Å; and by (3) comparing the overall number of correctly predicted interacting residues in the docked complex to the X-ray complex (where interacting residues are also defined as residues located less than 4Å from the ligand).

### Small-molecule docking analysis

The resulting ligand poses of the known hPKR antagonists were analyzed to identify all ligand-receptor hydrogen bonds, charged interactions, and hydrophobic interactions.

The specific interactions formed between the ligand and binding site residues were quantified to determine the best scoring pose of each ligand (active and inactive). For each ligand pose, a vector indicating whether this pose forms a specific hydrogen bond and/or hydrophobic π interaction with each of the binding site residues was generated. The data were hierarchically clustered using the clustergram function of the bioinformatics toolbox in Matlab version 7.10.0.499 (R2010a). The pairwise distance between these vectors was computed using the Hamming distance method, which calculates the percentage of coordinates that differ. For a *m*-by-*n* data matrix X, which is treated as *m* (1-by-*n*) row vectors x1, x2, …, x*m*, the distance between the vector x*s* and x*t* is defined as follows:
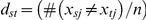
where # is the number of vectors that differ.

The poses of the virtual hits ligands were further filtered using structure-based constraints derived from analyzing the interactions between known PKR antagonists and the receptor, obtained in the known binders docking section of this work. The constraints included (1) an electrostatic interaction between the ligand and Glu119^2.61^, (2) at least one hydrogen bond between the ligand and Arg144^3.32^, and/or Arg307^6.58^, and (3) at least two hydrophobic interactions (π-π or π-cation) between the ligand and Arg144^3.32^ and/or Arg307^6.58^.

### Evolutionary selection analysis

Evolutionary selection analysis of the PKR subtypes' coding DNA sequences was carried out using the Selecton server (version 2.4) [46], [47]. The Selecton server is an on-line resource which automatically calculates the ratio (ω) between non-synonymous (Ka) and synonymous (Ks) substitutions, to identify the selection forces acting at each site of the protein. Sites with ω>1 are indicative of positive Darwinian selection, and sites with ω<1 suggest purifying selection. As input, we used the homologous coding DNA sequences of 13 mammalian species for each subtype, namely, human, rat, mouse, bovine, rabbit, panda, chimpanzee, orangutan, dog, gorilla, guinea pig, macaque and marmoset. We used the default algorithm options and the obtained results were tested for statistical significance using the likelihood ratio test, as implemented in the server.

## Results

### SAR analysis highlights molecular features essential for small-molecule antagonistic activity

A review of the literature revealed a group of non-peptidic compounds that act as small-molecule hPKR antagonists, with no apparent selectivity toward one of the subtypes [17], [18], [19], [20], [38]. The reported compounds have either a guanidine triazinedione or a morpholine carboxamide scaffold. We decided to perform structure-activity relationship (SAR) analysis of the triazine-based compounds, owing to the more detailed pharmacological data available for these compounds [17], [18], [19], [20].

SAR analysis of the reported molecules with and without antagonistic activity toward hPKR provides hints about the geometrical arrangement of chemical features essential for the biological activity. By comparing pairs of active and inactive compounds that differ in only one functional group, one can determine the activity-inducing chemical groups at each position.

**Figure 2:**
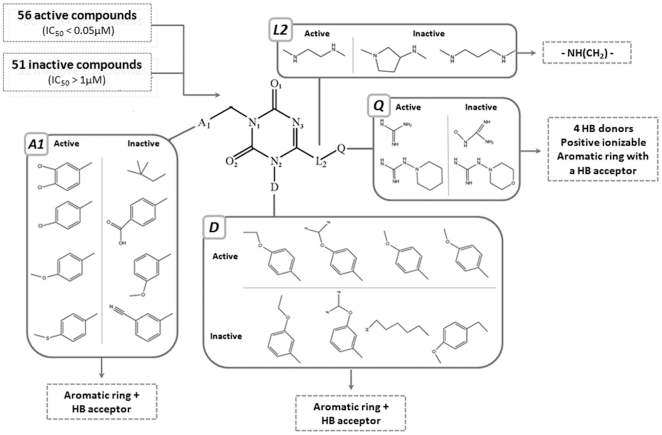
SAR analysis of small-molecule PKR antagonists identifies activity-determining chemical groups. The four variable positions in the scaffold – A1, D, L2, and Q, were compared in a dataset composed of 56 active compounds (IC_50_<0.05 µM) and 51 inactive compounds (IC_50_>1 µM) to determine the required chemical features at each position that elicit activity (a representative set is shown). These features are indicated in dashed boxes for each position. HB - hydrogen bond.

To this end, we constructed a dataset of 107 molecules identified by high-throughput screening. This included 51 molecules that we defined as inactive (Ca^2+^ mobilization IC_50_ higher than 1 µM), and 56 molecules defined as active (IC_50_ below 0.05 µM). All compounds share the guanidine triazinedione scaffold (see figure 2), which includes (a) a heterocyclic ring baring three nitrogen atoms and two oxygen atoms, and (b) a guanidine group, which is attached to the main ring by a linker (position Q in figure 2).

Where possible, the dataset was divided into pairs of active and inactive molecules that differ in only one functional group. This resulted in 13 representative pairs of molecules that were used to determine which specific chemical features in these molecules are important for antagonistic activity, in addition to the main triazine ring and guanidine group. As shown in figure 2, the four variable positions in the scaffold - A1, D, L2, and Q, were compared among the 13 pairs, and the activity-facilitating chemical groups at each position were determined. These include the following features:

1. Positions A1 and D require an aromatic ring with a hydrogen bond acceptor in position 4 of the ring.
2. Position L2 may only accept the structure -NH(CH_2_)-.
3. Position Q may include up to four hydrogen bond donors, a positive ionizable feature, and an aromatic ring bearing a hydrogen bond acceptor.

In conclusion, the SAR analysis revealed 2D chemical features in the molecules, which may be important for receptor binding and activation. Next, these features will be used to generate ligand-based pharmacophore models for virtual screening (next section) and in docking experiments to determine the plausible ligand-receptor contacts (see below).

### Ligand-based virtual screening for novel PKR binders

To identify novel potential hPKR binders, we utilized a ligand-based procedure in which molecules are evaluated by their similarity to a characteristic 3D fingerprint of known ligands, the pharmacophore model. This model is a 3D ensemble of the essential chemical features necessary to exert optimal interactions with a specific biological target and to trigger its biological response. The purpose of the pharmacophore modeling procedure is to extract these chemical features from a set of known ligands with the highest biological activity. The two most potent (IC_50_<0.02 µM for intracellular Ca^2+^ mobilization) hPKR antagonists were selected from the dataset described in the previous section, to form the training set (compounds 1 and 2, figure S3). In addition, we also incorporated data from a third compound published recently (compound 3 in figure S3), to ensure good coverage of the available chemical space [38].

**Figure 3:**
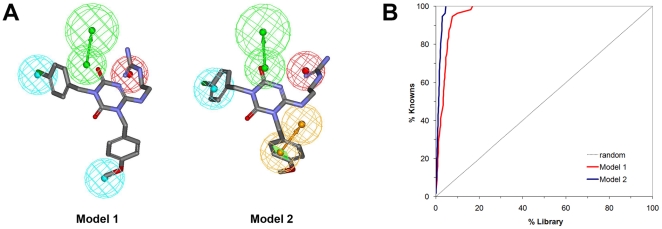
Ligand-based pharmacophore models recapture the known binders. (A) ligand-based four-feature pharmacophores used for virtual screening, with mapping of a known active small-molecule antagonist used for constructing the pharmacophores. The pharmacophores are represented as tolerance spheres with directional vectors where applicable. Green spheres represent hydrogen bond acceptors, red - positive ionizable, light blue – hydrophobic, and orange - aromatic ring. (B) ROC curve demonstrating the enrichment achieved following ligand-based pharmacophore mapping of 56 known active PKR antagonists and 5909 random molecules obtained from the ZINC database. Known actives are significantly enriched by both pharmacophore hypotheses.

The HipHop algorithm [37] was used to generate common features of pharmacophore models. This algorithm generated 10 different models, which were first tested for their ability to identify all known active hPKR triazine-based antagonists (data not shown). During the pharmacophore generation and analysis procedure, we also projected the knowledge generated during our 2D SAR analysis onto the 3D pharmacophore models, and chose those that best fit the activity-facilitating chemical features identified in the 2D SAR analysis previously described. The two best models, which recaptured the highest number of known active hPKR binders and included all required 2D features deduced from the SAR analysis, were chosen for further analysis. The 3D spatial relationship and geometric parameters of the models are presented in figure 3A. Both models share a positive ionizable feature and a hydrogen bond acceptor, corresponding to the N3 atom and O1 atoms on the main ring, respectively (figure 2). However, the models vary in the degree of hydrophobicity tolerated: model 2 is more restrictive, presenting one aromatic ring feature and one hydrophobic feature, whereas model 1 is more promiscuous, presenting two general hydrophobic features. The aromatic/hydrophobic features correspond to positions A1 and D of the scaffold (figure 2). Figure 3A also shows the mapping of one of the training set molecules onto the pharmacophore model. All four features of both models are mapped well, giving a fitness value (FitValue) of 3.602 and 3.378 for hypotheses 1 and 2, respectively. The fitness value measures how well the ligand fits the pharmacophore. For a four-feature pharmacophore the maximal FitValue is 4.

Next, we performed an enrichment study to ultimately evaluate the pharmacophore model's performance. Our aim was to verify that the pharmacophores are not only able to identify the known antagonists, but do so specifically with minimal false positives. To this end, a dataset of 56 known active hPKR small-molecule antagonists was seeded in a library of 5909 random molecules retrieved from the ZINC database [39]. The random molecules had chemical properties (such as molecular weight and formal charge), similar to the known PKR antagonists, to ensure that the enrichment is not simply achieved by separating trivial chemical features.

Both models successfully identified all known compounds embedded in the library. The quality of mapping was assessed by generating receiver operating characteristic (ROC) curves for each model (figure 3B), taking into consideration the ranking of fitness values of each virtual hit. The plots provide an objective, quantitative measure of whether a test discriminates between two populations. As can be seen from figure 3B, both models perform extremely well, generating almost a perfect curve. The difference in the curves highlights the difference in pharmacophore stringency. The stricter pharmacophore model 2 (which has an aromatic ring feature instead of a hydrophobic feature) performs best in identifying a large number of true positives while maintaining a low false positive rate. Thus, we used model 2 in the subsequent virtual screening experiments. Note that it is possible that some of the random molecules that were identified by the pharmacophore models, and received fitness values similar to known antagonists, may be potential hPKR binders. A list of these ZINC molecules is available in table S1. These compounds differ structurally from the known small-molecule hPKR antagonists because the maximal similarity score calculated using the Tanimoto coefficient, between them and the known antagonists, is 0.2626 (compounds that have Tanimoto coefficient values >0.85 are generally considered similar to each other).

This analysis revealed that the ligand-based pharmacophore models can be used successfully in a VLS study and that they can identify completely different and novel scaffolds, which nevertheless possess the required chemical features.

### hPKR1 as a potential off-target of known drugs

**Figure 4:**
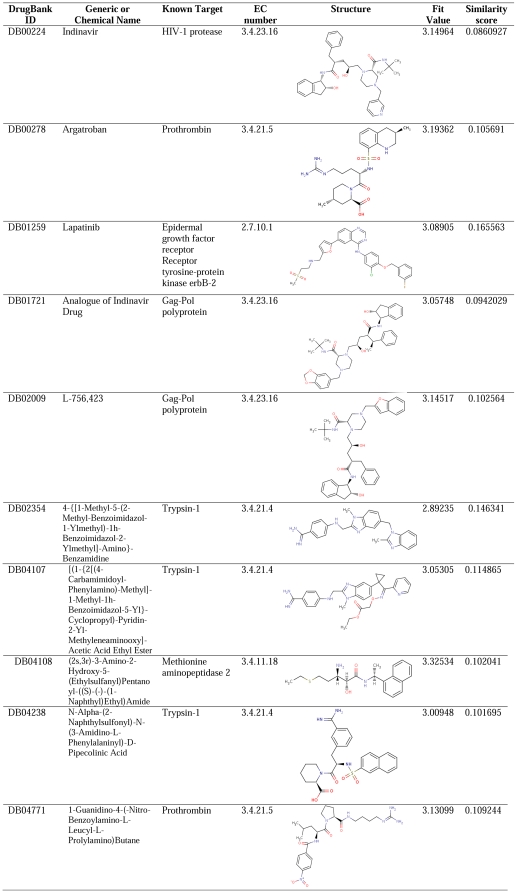
Final hits retrieved from virtual screening.

Recent work by Keiser and colleagues [48] utilized a chemical similarity approach to predict new targets for established drugs. Interestingly, they showed that although drugs are intended to be selective, some of them do bind to several different targets, which can explain drug side effects and efficacy, and may suggest new indications for many drugs. Inspired by this work, we decided to explore the possibility that hPKRs can bind established drugs. Thus, we applied the virtual screening procedure to a dataset of molecules retrieved from the DrugBank database (release 2.0) [40]. The DrugBank database [40] combines detailed drug (chemical, pharmacological, and pharmaceutical) data with comprehensive drug target (sequence, structure, and pathway) information. It contains 4886 molecules, which include FDA-approved small-molecule drugs, experimental drugs, FDA-approved large-molecule (biotech) drugs and nutraceuticals. As a first step in the VLS procedure, the initial dataset was pre-filtered, prior to screening, according to the average molecular properties of known active compounds ± 4SD. The pre-filtered set consisted of 432 molecules that met these criteria. This set was then queried with the pharmacophore, using the 'ligand pharmacophore mapping' module in DS2.5 (Accelrys, Inc.). A total of 124 hits were retrieved from the screening. Only those hits that had FitValues above a cutoff defined according to the pharmacophores' enrichment curve, which identifies 100% of the known antagonists, were further analyzed, to ensure that compatibility with the pharmacophore of the molecules selected is as good as for the known antagonists. This resulted in 10 hits with FitValues above the cutoff (see figure 4). These include 3 FDA-approved drugs and 7 experimental drugs. All these compounds target enzymes, identified by their EC numbers (corresponding to the chemical reactions they catalyze): most of the targets are peptidases (EC 3.4.11, 3.4.21 and 3.4.23), including aminopeptidases, serine proteases, and aspartic endopeptidases, and an additional single compound targets a receptor protein-tyrosine kinase (EC 2.7.10). The fact that only two classes of enzymes were identified is quite striking, in particular, when taking into account that these two groups combined represent only 2.6% of the targets in the screened set. This may indicate the intrinsic ability of hPKRs to bind compounds originally intended for this set of targets. The calculated similarity between the known hPKR antagonists and the hits identified using the Tanimoto coefficients is shown in figure 4: the highest similarity score was 0.165563, indicating that the identified hits are dissimilar from the known hPKR antagonists, as was also observed for the ZINC hits (see Table S1). Interestingly, when calculating the structural similarity within the EC3.4 and 2.7.10 hits, the highest value is 0.679, indicating consistency in the ability to recognize structurally diverse compounds (see figure S4).

To predict which residues in the receptor may interact with the key pharmacophores identified in the SAR analysis previously mentioned, and to assess whether the novel ligands harboring the essential pharmacophors fit into the binding site in the receptor, we carried out homology modeling and docking studies of the known and predicted ligands.

### Molecular Modeling of hPKR1 predicts the small-molecule binding site in the typical TM-bundle site of Family A GPCRs

As a first step in analyzing small-molecule binding to hPKRs, we generated homology models of the two subtypes, hPKR1 and hPKR2. The models were built using the I-Tasser server [29]. These multiple-template models are based on X-ray structures of bovine Rhodopsin (PDB codes: 1L9H) [49], the human β2-adrenergic receptor (2RH1) [50], and the human A_2A_-adenosine receptor (3EML) [51]. The overall sequence identity shared between the PKR subtypes and each of the three templates is approximately 20%. Although this value is quite low, it is similar to cases in which modeling has been applied, and it satisfactorily recaptured the binding site and binding modes [52]. Furthermore, the sequence alignment of hPKRs and the three template receptors are in good agreement with known structural features of GPCRs (figure S1). Namely, all TM residues known to be highly conserved in family A GPCRs [33] (N^1.50^, D^2.50^, R^3.50^, W^4.50^, P^5.50^, P^6.50^) are properly aligned. The only exception is the NP^7.50^xxY motif in TM7, which aligns to NT^7.50^LCF in hPKR1.

**Figure 5:**
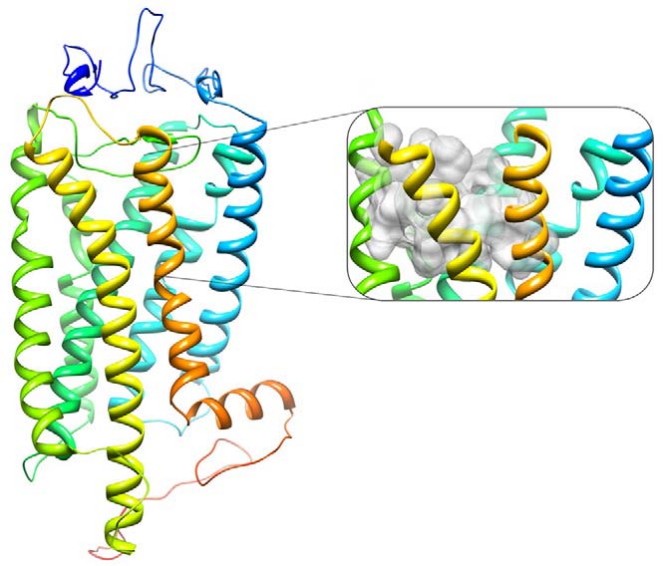
Homology model of hPKR1. The model is viewed perpendicular to the plasma membrane, with the extracellular side of the receptor shown on top, and the intracellular side shown on the bottom of the figure. The structure is colored from the N (blue) to the C (orange) terminal amino acid sequence. The insert shows the 7TM-bundle allosteric small-molecule binding site, predicted by the QSite Finder server. The binding site is located among TMs 3,4,5,6, and 7.

The initial crude homology model of hPKR1, obtained from I-TASSER, was further refined by energy minimization and side chain optimization. Figure 5 shows the general topology of the refined hPKR1 model. This model exhibits the major characteristics of family A GPCRs, including conservation of all key residues, and a palmitoylated cysteine in the C terminal tail, which forms a putative fourth intracellular loop. Also, similarly to family A GPCR X-ray structures, a conserved disulfide bridge connects the second extracellular loop (ECL2) with the extracellular end of TM3, formed between Cys217 and Cys137, respectively. However, both extracellular and intracellular loops are not very likely to be modeled correctly, due to their low sequence similarity with the template structures, and the fact that loop configurations are highly variable among GPCR crystal structures [42]. The emerging consensus in the field is that these models perform better in docking and virtual screening with no modeled loops at all than with badly modeled loops [43], [44], [45]. We therefore did not include the extracellular and intracellular loops in the subsequent analysis.

Overall, our hPKR1 model has good conservation of key features shared among family A GPCR members. Conservation of this fold led us to hypothesize that hPKRs possess a 7TM-bundle binding site capable of binding drug-like compounds, similar to the well-established TM bundle binding site typical of many family A GPCRs [25]. This is in addition to a putative extracellular surface binding site, which most likely binds the endogenous hPKR ligands, which are small proteins. Several synthetic small-molecule hPKR antagonists have been recently reported [17], [18], [19], [20], [38]. We hypothesized that these small molecules will occupy a pocket within the 7TM bundle [23], [53].

To identify the potential locations of a small-molecule-TM binding site, we first mapped all receptor cavities. We then utilized two energy-based methods, namely, Q-SiteFinder [35] and SiteHound [36], to locate the most energetically favorable binding sites by scanning the protein structure for the best interaction energy with different sets of probes. The most energetically favorable site identified by the two methods overlaps; it is located in the upper part of the TM bundle, among TMs 3,4,5,6, and 7. The position of the identified pocket is shown in the insert in Figure 5.

According to the structural superposition of the hPKR1 model on its three template structures, the predicted site is similar in position to the well-established TM-bundle binding site of the solved X-ray structures [54], [55]. Furthermore, specific residues lining these pockets, which are important for both agonist and antagonist binding by GPCRs [25], are well aligned with our model (figure S2).

Comparing the identified TM-bundle binding site between the two subtypes revealed that they are completely conserved, except for one residue in ECL2 - Val207 in hPKR1, which is Phe198 in hPKR2. Figure S5 presents a superposition of the two models, focusing on the binding site. This apparent lack of subtype specificity in the TM-bundle binding site is in agreement with the lack of specificity observed in activity assays of the small-molecule triazine-based antagonists [17], which could suppress calcium mobilization following Bv8 (a PK2 orthologue) stimulation to the same degree, in hPKR1 and hPKR2 transfected cells [17].

We therefore will focus mainly on hPKR1 and will return to the issue of subtype specificity in the Discussion.

### Docking of known small-molecule antagonists to hPKR1 binding site and identification of important interacting residues

To understand the mechanistic reasons for the need of particular pharmacophores for ligands activity, one has to look for interactions between the ligands and the receptor.

As a preliminary step, we performed a validation study, aimed at determining whether our modeling and docking procedures can reproduce the bound poses of representative family A GPCR antagonist-receptor crystallographic complexes. We first performed redocking of the cognate ligands carazolol and cyanopindolol, back to the X-ray structures from where they were extracted and from which the loops were deleted. The results indicate that the docking procedure can faithfully reproduce the crystallographic complex to a very high degree (figure S6 – A–C); with excellent ligand RMSD values of 0.89–1.2Å between the docked pose and the X-ray structure (see table S2), in accordance with similar previous studies [44], [56], [57]. The redocking process could also reproduce the majority of heavy atomic ligand-receptor contacts observed in the X-ray complex and more generally, the correct interacting binding site residues and specific ligand-receptor hydrogen bonds, despite docking to loopless structures. Next, we built homology models of β1adr and β2adr and performed docking of the two antagonists into these models to examine the ability of homology modeling, combined with the docking procedure, to accurately reproduce the crystal structures. As can be seen from figure S6 and from the ligand RMSD values in table S2, the results can reproduce the correct positioning of the ligand in the binding site, and at least part of the molecule can be correctly superimposed onto the crystallized ligand, although the resulting RMSD values are above 2Å. The overall prediction of interacting binding site residues is good, correctly predicting 47–66% of the interactions (see Table S2).

We therefore performed molecular docking of the small-molecule hPKR antagonist dataset to the predicted hPKR1 allosteric 7TM-bundle binding site, to explore the possible receptor-ligand interactions.

The set of 56 active and 51 inactive small-molecule antagonists was subjected to flexible ligand – rigid receptor docking to the hPKR1 model using LigandFit (as implemented in DS2.5, Accelrys, Inc.) [34]. For each compound the 50 best energy conformations were generated and docked into the binding site, resulting in an average of 250 docked poses for each molecule.

**Figure 6:**
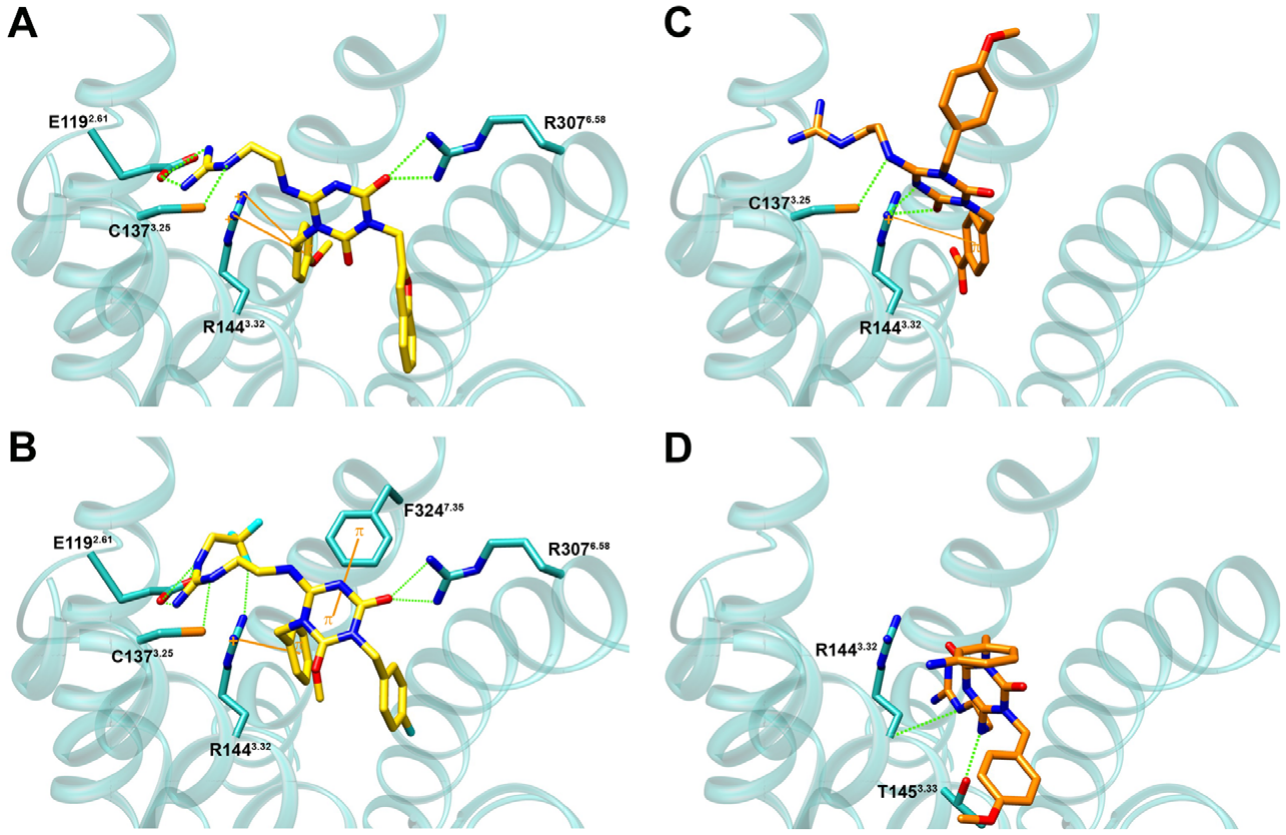
Interaction of receptor residues with active and inactive antagonists in the allosteric hPKR1 binding site. Representative docked poses of two known active compounds (A, B, IC_50_<0.05 µM), and two inactive compounds (C, D, IC_50_>1 µM) to the hPKR1 binding site. The active compounds are denoted by yellow sticks and the inactive ones as orange sticks. Interacting receptor residues are denoted by cyan sticks and labeled. Hydrogen bonds are denoted by dashed green lines and π-cation or π-π interactions are denoted by orange lines.

The final ligand poses for each molecule were selected based on the highest LigScore1 docking score, since no experimental data regarding possible ligand contacting residues was available. The best scoring docking poses were analyzed visually for features that were not taken into account in the docking calculation, such as appropriate filling of the binding site – such that the compound fills the binding site cavity, and does not "stick out". Specific ligand-receptor interactions were monitored across all compounds. Figure 6 shows representative docked poses of two active (A,B) and two inactive compounds (C,D). As shown, the active molecules adopt a confirmation that mainly forms interactions with TMs 2, 3, and 6, such that the ligand is positioned in the center of the cavity, blocking the entry to it and adequately filling the binding site, as described. In contrast, the inactive small molecules are apparently incapable of simultaneously maintaining all of these contacts, and are positioned in different conformations that mostly maintain interactions with only some of the TMs mentioned.

**Figure 7:**
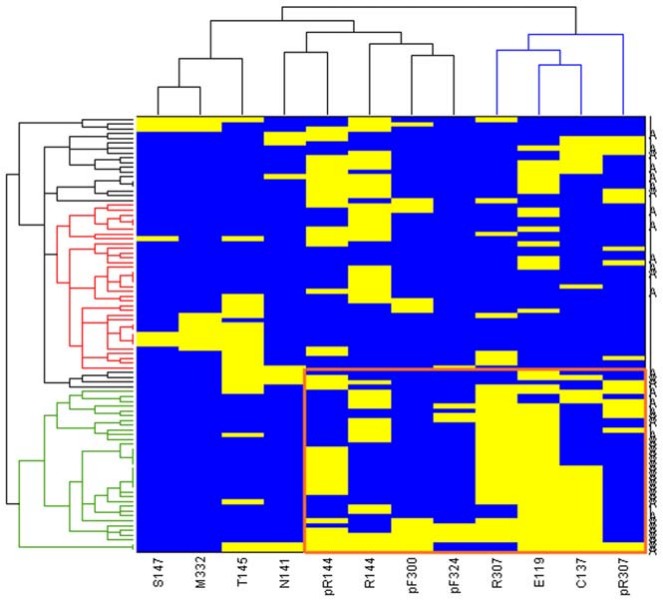
Clustering of hydrogen bond, charged, and π interaction patterns in the docked compounds correspond to activity level. Receptor residues forming the interactions are specified on the bottom of the clustergram. Residues denoted by p form π interactions (either π-cation or π- π stacking). Yellow represents a ligand-receptor contact. The sub-tree formed predominantly by the active ligands is in green, and the one formed predominantly by inactive ligands is in red. The “A” on the right side denotes active compounds. The specific pattern formed by the active compounds is boxed in orange. The black subtrees are mixed.

For the active compounds, the most prevalent interaction is observed between the ligand and residues Arg144^3.32^ and Arg307^6.58^, either through a hydrogen bond or a π-cation interaction. The active ligands interact with at least one of these two residues. In addition, an electrostatic interaction was observed between the active ligands and Glu119^2.61^ (as seen from figure 6A, B). To quantify this observation, the specific interactions formed (HB, charged, π-π and π-cation) were monitored across all the best scoring poses of the docked ligands (active and inactive), and the results, which represent the number of specific contacts formed between each ligand and all polar/hydrophobic binding site residues, were clustered (figure 7).

As shown, the hierarchical structure obtained from the clustering procedure of receptor-ligand contacts only, clearly separates the compounds into sub-trees that correspond to the experimental active/inactive distinction. In the active sub-tree, the ligands form a charged interaction with Glu119^2.61^, and interact mainly with Cys137^3.25^, Arg144^3.32^, and Arg307^6.58^. In contrast, in the inactive sub-tree, the molecules still form interactions with Arg144^3.32^ to some extent, but the interactions with Glu119^2.61^, Cys137^3.25^, and Arg307^6.58^ are drastically reduced, and instead some of the ligands interact with Thr145^3.33^ and Met332^7.47^. In addition, some of the active ligands form either specific interactions or van der Waals contacts with Asn141^3.29^, Phe300^6.51^, and Phe324^7.39^.

All of these positions have been shown experimentally to be important for ligand binding in different family A GPCRs members, ranging from aminergic (such as the β2-adrenergic receptor) to peptide receptors (such as chemokine receptors) [25].

**Figure 8:**
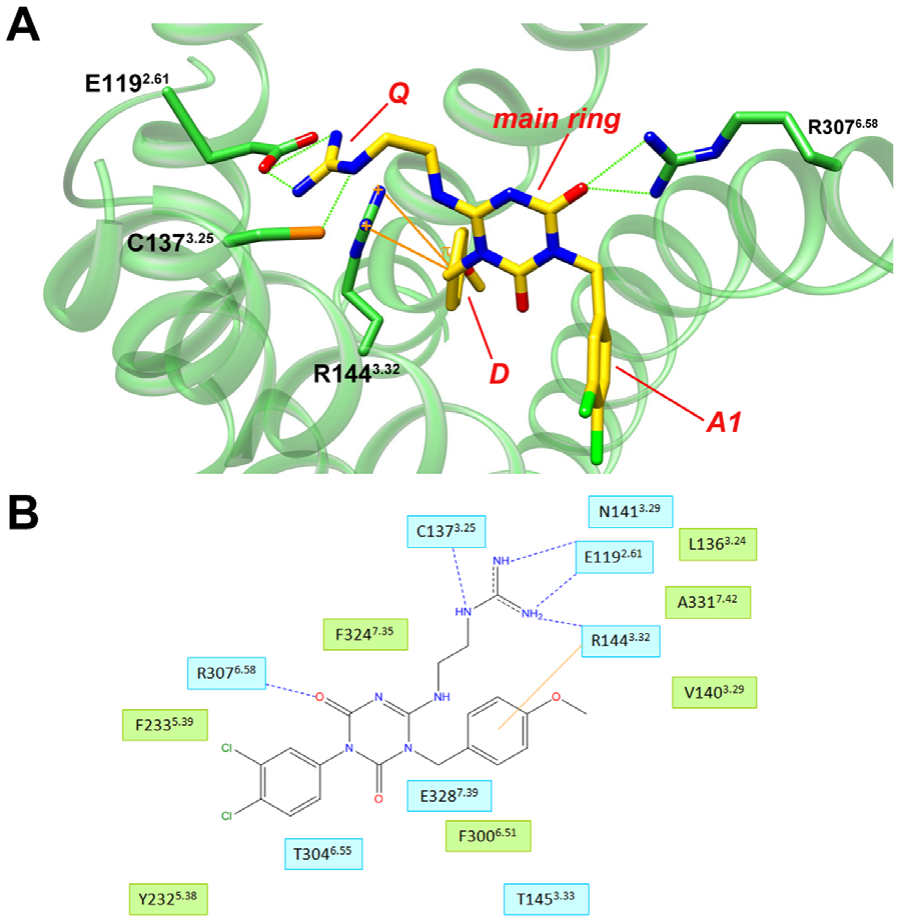
The essential activity-determining groups of the known binders form specific interactions with the receptor. (A) An example of the ligand's chemical properties, which are important for its activity, identified in the SAR analysis, and how they interact with receptor residues. An active molecule is shown docked into the hPKR1 binding site. The activity-related chemical groups are indicated in red. The ligand is denoted by yellow sticks. Interacting receptor residues are shown as green sticks and labeled. Hydrogen bonds are denoted by dashed green lines, π-cation interactions are denoted by orange lines. (B) Schematic 2D representation of ligand-receptor interactions. The residues shown have at least one atom within 4Å of the ligand. Blue lines indicate hydrogen bonds and orange lines indicate hydrophobic interactions. Residues shaded in green are involved in van der Waals interactions. Residues involved in hydrogen bonds, charge, or polar interactions are shaded in cyan.

In general, the functional groups in the scaffold, which were identified in our SAR analysis as being important for antagonist activity, form specific interactions within the binding site (figure 8). Namely, the main triazine ring of the scaffold forms hydrogen bonds through its O and N atoms and π-cation interactions. The two aromatic rings form π-cation interactions and hydrogen bonds through the O/F/Cl atoms at position 4 of the ring, and the positive charge at position Q and hydrogen bond donors interact with residues from helices 2, 3, and 6, predominantly, Glu119^2.61^ and Arg144^3.32^, and Arg307^6.58^, as described above. The compatibility of the SAR data with the docking results supports the predicted binding site and modes, and provides a molecular explanation of the importance of particular pharmacophores in the ligand.

The positions predicted to specifically bind essential functional groups in the ligands (mainly Glu119^2.61^, Arg144^3.32^, and Arg307^6.58^) can be mutated in future studies, to confirm their role in ligand binding inside the predicted TM-bundle cavity, as recently applied to other GPCRs [58] and summarized in [25].

### Docking of virtual hits to the hPKR1 model suggests potential binders

Next, the 10 molecules identified through ligand-based virtual screening of the DrugBank database were docked to the hPKR1 homology model. All docking experiments were performed using LigandFit, as described in the previous section. However, here the analysis was more strict: the resulting docked poses of each molecule were post-processed using structure-based filters derived from the analysis of ligand-receptor interactions formed between the known small-molecule antagonists and receptor residues (see Materials and Methods for details) and were not only selected based on the highest docking score. The underlying hypothesis is that the same interactions are perused by the potential ligands as by the known antagonists. Selected poses of all 10 molecules successfully passed this procedure. All poses were visually examined by checking that they adequately fill the binding site and form the desired specific interactions. All 10 molecules successfully passed this analysis and were considered as candidate compounds that may serve as potential hPKR binders.

**Figure 9:**
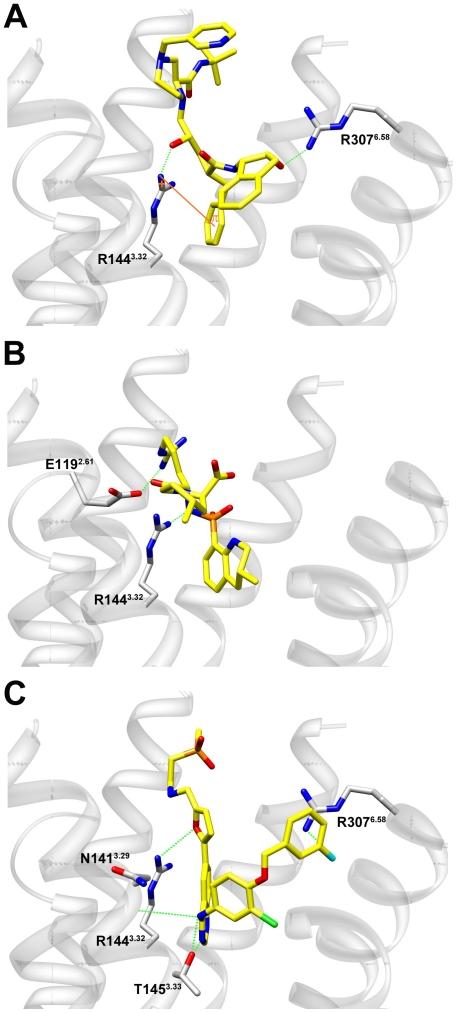
Docking of potential PKR binders identified through VLS, to the hPKR1 binding site. The proposed docked conformations of (A) Indinavir, (B) Argatroban, and (C) Lapatinib are shown. The ligands are denoted by yellow sticks. Interacting receptor residues are denoted by gray sticks and labeled. Hydrogen bonds are denoted by dashed green lines, and π-cation interactions are denoted by orange lines.

Next, we focused on a representative of the three FDA-approved hits, which we identified as potential ligands for hPKRs, namely, Indinavir, Argatroban, and Lapatinib. Figure 9 shows representative examples of docking of Indivavir, Argatroban, and Lapatinib to the hPKR1 binding site. As shown, the compounds adequately fill the binding site and are predicted to form specific interactions with residues found to be important for binding of the known hPKR antagonists, namely, charged interaction with Glu119^2.61^, and hydrogen bonds and/or stacking interactions with Arg144^3.32^ and Arg307^6.58^. These compounds also form interactions with additional binding site residues, which interact with the known binders (see figure 7).

Each of the compounds is widely used in the clinic, and provides well-tested and safe compounds that may also exert their actions via hPKRs. The potential cross-reactivity of one such candidate drug, Indinavir, is further addressed in the Discussion.

## Discussion

Prokineticin receptor (PKR) subtypes 1 and 2 are novel members of family A GPCRs. Prokineticins and their receptors play important roles under various physiological conditions, and blocking PKRs may serve as a therapeutic tool for various pathologies, including acute pain, circadian rhythm disturbances, inflammation, and cancer.

In this study, we extracted essential functional groups from small-molecule PKR antagonists that were previously reported, using structure-activity relationship analysis, and we used them in a virtual screening procedure. Consequently, we were able to identify several potential PKR ligands with novel scaffolds. Interestingly, the virtual hits included several HIV protease inhibitors that are discussed next in terms of known side effects and potential new indications of these drugs. Computational docking of known ligands to the multiple-template 3D model of a PKR's structure enabled us to predict ligand-receptor contacts and provided a structural explanation of the importance of the chemical features we obtained from the analysis of known PKR binders.

### Homology modeling of the hPKR subtypes and docking of known small-molecule antagonists

In this study we modeled the 3D structure of the hPKR subtypes and explored the interactions formed between hPKR1 and small-molecule binders. Our computational analysis revealed that hPKR1 is predicted to possess a TM-bundle binding site, capable of binding small-molecule ligands, similarly to other GPCR family A members, such as the aminergic receptors. This occurs despite the fact that the receptors' endogenous ligands are relatively large proteins, which most likely bind the extracellular surface of the receptors. The latter is demonstrated in experimental data on Kallmann syndrome mutations. Kallmann syndrome is a human disease characterized by the association of hypogonadotropic hypogonadism and anosmia. Several loss-of-function mutations in the human PKR2 gene have been found in Kallmann patients [45]. Among them is the p.Q210R mutation in ECL2 (corresponding to Q219 in hPKR1), which completely abolishes native ligand binding and has no affinity for the orthologue ligand MIT1 (Mamba intestinal toxin 1, which shares 60% sequence identity with PK2, and contains the essential N-terminal motif AVITGA) [59]. Existence of both an orthosteric extracellular binding site capable of binding small proteins and an allosteric TM binding site was already shown in family A GPCRs. For example, the melanin-concentrating hormone receptor (MCHR), for which the endogenous ligand is a peptide, also binds small-molecule antagonists in its TM-bundle cavity [60], [61].

The predicted TM-bundle site is identical between the two hPKR subtypes, except for one residue in ECL2 (Val207 in PKR1 corresponding to Phe198 in PKR2). Since this is a hydrophobic residue in both receptors, its side chain will probably face the TM cavity and not the solvent. Indeed, the residue was modeled to face the TM cavity and was predicted by the energy-based methods to be part of the TM-bundle binding site. If specific binders are pursued in the future, this, albeit minor, difference between two hydrophobic amino acids might be targeted.

Through docking experiments of the known hPKR antagonists, we have identified important residues that interact at this site, namely, Glu119^2.61^, Arg144^3.32^, and Arg307^6.58^. These residues form specific interactions with the chemical features of the ligand that we found in our SAR analysis to be essential for the molecules' antagonistic activity. Specifically, Arg144^3.32^ is analogous to Asp113^3.32^ of the β2-adrenergic receptor, which is an experimentally established receptor interaction site for both agonists and antagonists [62]. This position has also been shown to be important for ligand binding in many other family A GPCRs as well as in other branches of the GPCR super-family, such as the bitter taste receptors (summarized in [25]). This position is highly conserved *within* different family A GPCRs subfamilies, but it is divergent among these subfamilies, for example, an Asp in the aminergic receptors, compared with a Thr in hormone protein receptors. It was therefore assumed that the position may play a role in specific ligand binding within certain subfamilies [55]. Similarly, we suggest that although the residue type is divergent between the different subfamilies (for example, a positive Arg in the Prokineticin receptors compared with a negative Asp in aminergic receptors), its importance in ligand binding in such diverse receptors may be due to its spatial location in the TM-bundle binding site. In addition, Arg307^6.58^ is analogous to Tyr290^6.58^ of the GnRH receptor, which was found to be important for binding the GnRH I and GnRH II peptide ligands [63]. The equivalent residue at position 6.58 is also suggested, by mutagenesis studies, to play an important role in ligand binding and/or receptor activation of other peptide GPCRs, such as the NK2 tachykinin receptor [64], the AT_1A_ angiotensin receptor [65], and the CXCR1 chemokine receptor [66]. Moreover, in the recent crystallographic X-ray structure of the CXCR4 chemokine receptor bound to a cyclic peptide antagonist, a specific interaction between position 6.58 and the peptide was observed [67]. Hence, position 6.58 may serve as a common position for the binding of both peptides (such as the endogenous ligands PK1 and PK2) and small-molecule ligands.

Finally, in our analysis position 2.61, which is occupied by a Glutamic acid in hPKRs, was found to be essential for antagonist binding, since an electrostatic interaction may be formed between this negatively charged residue and the positive charge on the ligand. This may explain the need for the positive charge on the known small-molecule antagonists, which was indeed deduced from the structure-activity analysis. The ligand's positive charge may interact with the negatively charged residue in receptor position 2.61, which was also shown to be important in ligand binding in the dopamine receptors [55].

In summary, the observed interactions reinforce the predicted putative binding site and may support the concept that family A GPCRs share a common small-molecule binding pocket inside the TM cavity, regardless of the nature of their cognate ligand.

Docking of ligands to a single experimental or model structure of a GPCR receptor has been shown to reproduce the binding mode of the ligands in several cases [44], [68], [69], to enrich known ligands in structure-based virtual screening campaigns [57], [70], and to rationalize specificity profiles of GPCR antagonists [71] and thus was the approach taken here.

In several non-GPCR cases, good docking results have been reported using multiple receptor conformations [72]. Such an approach was successful for a sequence identity range of 30–60% between models and available templates [73].

Though GPCR homology models typically have a lower sequence identity to their potential templates, using ensembles of multiple homology models or of a perturbed X-ray structure may nevertheless be a viable approach, as was recently reported [74], [75], [76]. Current breakthroughs in X-ray structure determination of GPCRs will enable systematic testing of the most appropriate receptor structure representation and of docking performance, against the benchmark of experimental structures.

### Identification of potential novel hPKR binders

Our study used SAR of known hPKR binders to identify novel potential binders of hPKR1, and highlighted possible 'off-target' effects of FDA-approved drugs. Interestingly, the novel candidates share little structural chemical similarity with the known hPKR binders but share the same pharmacophores and similar putative interactions within the TM-bundle binding site. Such a "scaffold hopping" result is common and is often sought after in drug discovery. The term is based on the assumption that the same desired biological activity may be achieved by different molecules that maintain some of the essential chemical features as the template molecule, i.e., the molecule possesses the desired biological activity on the target, but is structurally dissimilar otherwise. Scaffold hopping is required, for instance, when the central scaffold is involved in specific interactions with the target, and changing it may lead to improved binding affinity. One example of successful scaffold hopping, resulting in a structurally diverse structure, is the selective D2 and D3 dopamine receptor agonist Quinpirole [77].

The newly identified potential cross-reactivity may have two implications – it might explain the side effects of these drugs (as discussed next), and it might also suggest novel roles for these drugs as potential hPKR inhibitors. One such example of potential cross-reactivity identified through our VLS procedure is Indinavir.

Indinavir sulfate is a hydroxyaminopentane amide and a potent and specific FDA-approved inhibitor of the HIV protease. Indinavir acts as a competitive inhibitor, binding to the active site of the enzyme, since it contains a hydroxyethylene scaffold that mimics the normal peptide linkage (cleaved by the HIV protease) but which itself cannot be cleaved. Thus, the HIV protease cannot perform its normal function - proteolytic processing of precursor viral proteins into mature viral proteins. Specific adverse effects associated with Indinavir include hyperbilirubinaemia and cutaneous toxicities [78], [79], accelerated atherosclerosis, and an increased rate of cardiovascular disease [80]. Protease inhibitors may cause cardiovascular disease by inducing insulin resistance, dyslipidemia, or by endothelial dysfunction.

A study of the effects of HIV protease inhibitors on endothelial function showed that in healthy HIV-negative subjects, Indinavir induced impaired endothelium-dependent vasodilation after 4 weeks of treatment owing to reduced nitric oxide (NO) production/release by the endothelial cells or reduced NO bioavailability [81]. HIV patients treated with Indinavir presented lower urinary excretion of the NO metabolite NO_3_ [82]. Wang et al. demonstrated that Indinavir, at a clinical plasma concentration, can cause endothelial dysfunction through eNOS (endothelial nitric oxide synthase) down-regulation in porcine pulmonary artery rings and HPAECs (human pulmonary arterial endothelial cells), and that endothelium-dependent relaxation of the vessel rings was also reduced following Indinavir treatment [83].

Endothelium-derived NO is the principal vasoactive factor that is produced by eNOS. Lin et al. showed that PK1 induced eNOS phosphorylation in bovine adrenal cortex-derived endothelial cells [14]. It has also been shown that PK1 suppressed giant contraction in the circular muscles of mouse colon, and that this effect was blocked by the eNOS inhibitor L-NAME. In vitro, PK1 stimulated the release of NO from longitudinal musclemyenteric plexus cultures [84]. We have found that PK1 treatment elevated eNOS mRNA levels in luteal endothelial cells. Cells were also treated in the presence of PI3/Akt pathway inhibitor, which caused a 20–40% reduction in eNOS levels (Levit and Meidan, unpublished data).

These opposing effects of Indinavir and PK1 on eNOS levels and NO production/release are compatible with the chemically based hypothesis arising from the current work, which suggests that Indinavir can bind to the hPKR subtypes by acting as a PKR antagonist. We suggest that this would subsequently reduce eNOS expression levels in endothelial cells and impair NO bioavailability, leading, at least partially, to the observed Indinavir side effects in HIV patients. This hypothesis should be explored experimentally in future studies to determine the possible binding of Indinavir to hPKRs and its subsequent effects.

The proposed hypothesis is in accordance with the concept of polypharmacology - specific binding and activity of a drug at two or more molecular targets, often across target boundaries. For example, ligands targeting aminergic family A GPCRs were also found to act on protein kinases [85]. These "off-target" drug actions can induce adverse side effects and increased toxicity. In contrast, there are also cases where the drug is a "magic shotgun", and its clinical effect results from its action on many targets, which in turn enhances its efficacy. For example, drugs acting through multiple GPCRs have been found to be more effective in treating psychiatric diseases such as schizophrenia and depression [86]. This concept was demonstrated by Keiser and colleagues [48] who utilized a statistics-based chemoinformatics approach to predict off-targets for ∼900 FDA-approved small-molecule drugs and ∼2800 pharmaceutical compounds. The targets were compared by the similarity of the ligands that bind to them. This comparison resulted in 3832 predictions, of which 184 were inspected by literature searches. Finally, the authors tested 30 of the predictions experimentally, by radioligand competition binding assays. For example, the α1 adrenergic receptor antagonist Doralese was predicted and observed to bind to the dopamine D4 receptor (both are aminergic GPCRs), and most interestingly, the HIV-1 reverse transcriptase inhibitor Rescriptor was found to bind to the histamine H4 receptor. The latter observation crosses major target boundaries. These two targets have neither an evolutionary or functional role nor structural similarity in common. However, some of the known side effects of Rescriptor treatment include painful rashes. This observation is similar to our findings of possible interactions of Indinavir and the other enzyme-targeting VLS hits with the PKR subtypes.

In summary, defining the selective and non-selective actions of GPCR targeting drugs will help in advancing our understanding of the drugs' biological action and the observed clinical effect, including side effects.

### Potential differences between the hPKR subtypes

Both subtypes are capable of binding the cognate ligands at approximately the same affinity [12]. Therefore, the diversification of cellular events following activation of the subtypes [16] is not likely to stem from the extracellular loop regions. This suggestion warrants further experimental investigation. Our study also suggests, in agreement with previous findings, that small-molecule antagonists are not likely to easily differentiate between the subtypes. This is because the TM-bundle small-molecule binding site identified in this study is identical in its amino acid composition for the two hPKR subtypes. Thus, an intriguing question arises: what molecular mechanisms are responsible for PKRs' differential signaling patterns?

The variation of protein amino acid composition in the extracellular and intracellular regions of PKRs is significant (represented as black-filled circles in Fig. 1). Moreover, analysis of the level of selection acting on the two PKR subtypes, by calculating the ratio between non-synonymous (Ka) and synonymous (Ks) substitutions [46], [47] predicted purifying selection for the transmembrane helices of both subtypes (figure S7). This analysis should be expanded in future studies, as PKR subtype sequences from additional species become available.

The variation in amino acid composition in the intracellular regions of the PKR subtypes may affect at least two signaling events: receptor phosphorylation by kinases and the receptors' coupling to G proteins. We therefore suggest that this region is most likely to be involved in differential signaling, as detailed next.

### Interaction with G proteins

Differential coupling of PKR subtypes to G proteins has been demonstrated experimentally (reviewed in [16]). Coupling of PKR1 to G_α11_ in endothelial cells induces MAPK and PI3/Akt phosphorylation, which promotes endothelial cell proliferation, migration and angiogenesis [11]. In cardiomyocytes, coupling of PKR1 to G_αq/11_ induces PI3/Akt phosphorylation and protects cardiomyocytes against hypoxic insult. In contrast, PKR2 couples to G_α12_ in endothelial cells, causing G_α12_ internalization and down-regulation of ZO-1 expression, leading to vacuolarization and fenestration of these cells. In cardiomyocytes, PKR2 acts through G_α12_ and G_αq/11_ coupling and increases cell size and sarcomere numbers, leading to eccentric hypertrophy [16]. Thus, sites of interactions with G-proteins may represent an additional factor affecting PKR subtype specificity.

### Receptor Phosphorylation

It is well established that GPCR phosphorylation is a complex process involving a range of different protein kinases that can phosphorylate the same receptor at different sites. This may result in differential signaling outcomes, which can be tailored in a tissue-specific manner to regulate biological processes [87]. We suggest that part of the differential signaling of PKR subtypes may be due to differential phosphorylation of the intracellular parts of the receptors. Namely, phospho-acceptor sites may be missing in one subtype or another, and analogous positions may be phosphorylated by different kinases due to variation in the positions surrounding the phospho-acceptor residue (which is conserved between subtypes), thus, changing the kinase recognition sequence [88]. Hence, using different combinations of kinases for each subtype results in different phosphorylation signatures. This phosphorylation signature translates to a code that directs the signaling outcome of the receptor. This may include two types of signaling events: (a) common phosphorylation events for both subtypes will mediate common regulatory features such as arrestin recruitment and internalization and (b) subtype-specific events will mediate specific signaling functions related to the specialized physiological role of the receptor subtype. Preliminary analysis using prediction tools for phosphorylation sites suggests that Thr178 (Thr169) in the second intracellular loop and Tyr365 (Gln356) in the cytoplasmic tail of hPKR1 (hPKR2) may represent subtype-specific phosphorylation-related sites (Levit, Meidan and Niv, unpublished data). Further experimental studies are required to elucidate the role of receptor phosphorylation in specific signaling events following activation of PKR subtypes.

### Conclusions

In conclusion, we have identified a small-molecule TM-bundle site that can accommodate the known small-molecule hPKR antagonists. Hence, it can be explored in the future for designing additional PKR-targeting compounds. The VLS procedure identified tens of compounds that are likely to affect hPKRs. Interestingly, FDA-approved drugs may also bind to these receptors, and in some instances, such as with Indinavir, this binding may provide a potential explanation for the drug's side effects. One residue in ECL2 is different between the two subtypes (Val207 in hPKR1 corresponding to Phe198 in PKR2), and several residues in the intracellular loops may affect phosphorylation. These residues may be exploited for designing subtype-specific pharmacological tools, to target different pathological conditions involving hPKRs.

## Supporting Information

Figure S1 **Structure-based multiple sequence alignment of modeled PKR subtypes and X-ray structures used as templates in the modeling procedure.** Alignment was generated by the TCoffee server. The most conserved residue in each helix is shaded yellow and is indicated by its Ballesteros-Weinstein numbering [33]. Identical residues are in red and similar residues are in blue. bRho - bovine Rhodopsin (PDB code:1L9H), hB2ADR - human β2-adrenergic receptor (2RH1), hA2AR - human A_2A_ adenosine receptor (3EML). The sequence of T4 lysozyme that was fused to the hB2ADR and hA2AR proteins to facilitate structure determination was removed prior to alignment, for clarity.
(TIF)

Figure S2 **Structural superposition of the PKR1 model and GPCR X-ray templates used for homology modeling.** All structures are shown in ribbon representation. PKR1 is in turquoise, human β2-adrenergic is in orange (A), bovine rhodopsin is in gold (B) and human A_2A_-adenosine receptor is in gray (C). (D) Superposition of the hPKR1 model and the β2-adrenergic receptor structure with emphasis on the TM-bundle binding site. The structures are shown in a view looking down on the plane of the membrane from the extracellular surface. Binding site residues experimentally known to be important for ligand binding are denoted as sticks and are labeled with Ballesteros-Weinstein numbering. The T4 lysozyme fusion protein was removed from the β2-adrenergic and the A_2A_-adenosine receptor structures, for clarity. Structural superposition was performed using the Matchmaker module in UCFS Chimera version 1.4.1.
(TIF)

Figure S3 **Structures of the three known PKR antagonists that were used as reference compounds for constructing ligand-based pharmacophore models.**
(TIF)

Figure S4 **Structural similarity between the identified VLS hits plotted as a heatmap.** The degree of similarity was calculated using the Tanimoto coefficient, as described in Methods, and ranges between 0 (completely dissimilar compounds) and 1 (identical compounds). Compounds with similarity values >0.85 are usually considered structurally similar. Color intensity corresponds to the similarity value according to the legend. The heatmap was prepared using Matlab version 7.10.0.499 (R2010a).
(TIF)

Figure S5 **Structural superposition of human PKR1 and PKR2 models.** Both structures are shown in ribbon representation, with hPKR1 in turquoise and hPKR2 in khaki. The insert shows a detailed view of the predicted transmembrane binding site, with side chains denoted as sticks. Structural superposition was performed using the Matchmaker module in UCFS Chimera version 1.4.1.
(TIF)

Figure S6 **Predicted binding modes of cognate ligands redocked into crystal structures and homology models.** (A) Cyanopindolol redocked to β1adr crystal structure (PDB code: 2VT4), (B) Carazolol redocked to β1adr crystal structure (2YCW), (C) Carazolol redocked to β2adr crystal structure (2RH1), (D) Cyanopindolol docked to β1adr homology model, (E) Carazolol docked to β1adr homology model and (F) Carazolol docked to β2adr homology model. The docked ligands are shown as green sticks. X-ray structures are represented as gray ribbons and the crystallized ligand is shown as gray sticks. In panels (D–F) the homology models are shown as gold ribbons.
(TIF)

Figure S7 **Measure of Ka/Ks ratio on the amino acid sequence of the PKR subtypes** **suggests positive selection acting only on PKR2.** Ka/Ks ratio (ω) representing the ratio of non-synonymous (Ka) to synonymous (Ks) nucleotide substitution rates was calculated for each site for the PKR subtypes. The ratio is plotted against the amino acid position for hPKR1 (A) and hPKR2 (B). Residues showing ω>1 are indicative of positive Darwinian selection, while residues showing ω<1 are indicative of purifying selection; the ratio for neutral selection is one (indicated on the graph by a red line). Significant positive selection (p = 0.001) was detected only for PKR2, by the likelihood ratio test, and is concentrated in the N-terminus and C-terminus domains.
(TIF)

Table S1 **Potential hits identified from the ZINC database.**
(DOC)

Table S2 **Ligand RMSD values and contact analysis for cognate ligand docking to** β**1adr and** β**2adr crystal structures and homology models.**
(DOC)
