# Supplementary figures and images for: Modeling of Human Prokineticin Receptors: Interactions with Novel Small-Molecule Binders and Potential Off-Target Drugs
Source: PLoS One. 2011 Nov 21;6(11):e27990. doi: 10.1371/journal.pone.0027990 (PMC3221691; doi:10.1371/journal.pone.0027990)

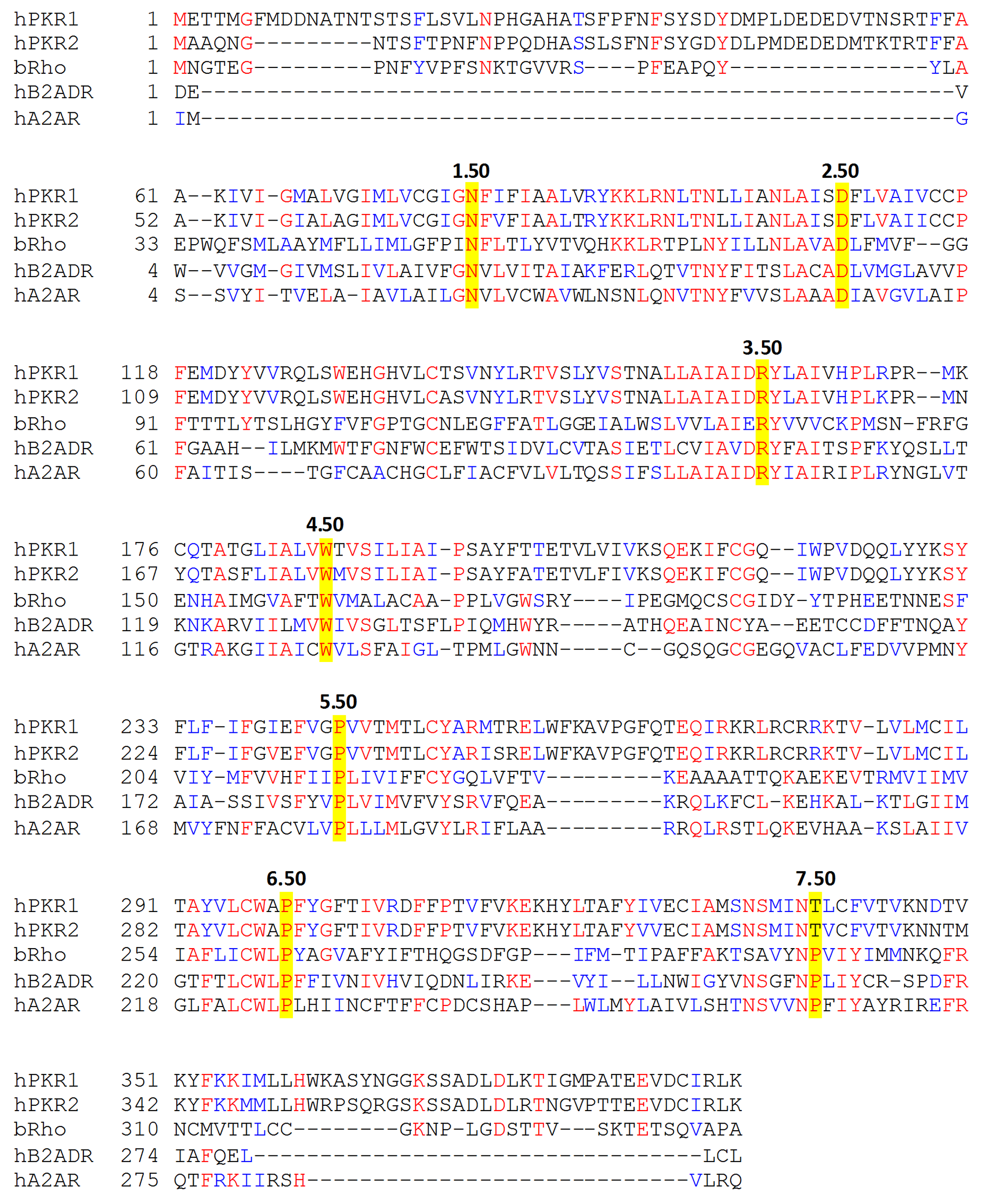

Supplement: Figure S1 — Structure-based multiple sequence alignment of modeled PKR subtypes and X-ray structures used as templates in the modeling procedure. Alignment was generated by the TCoffee server. The most conserved residue in each helix is shaded yellow and is indicated by its Ballesteros-Weinstein numbering [33]. Identical residues are in red and similar residues are in blue. bRho - bovine Rhodopsin (PDB code:1L9H), hB2ADR - human β2-adrenergic receptor (2RH1), hA2AR - human A2A adenosine receptor (3EML). The sequence of T4 lysozyme that was fused to the hB2ADR and hA2AR proteins to facilitate structure determination was removed prior to alignment, for clarity. (TIF) [file pone.0027990.s001.tif]

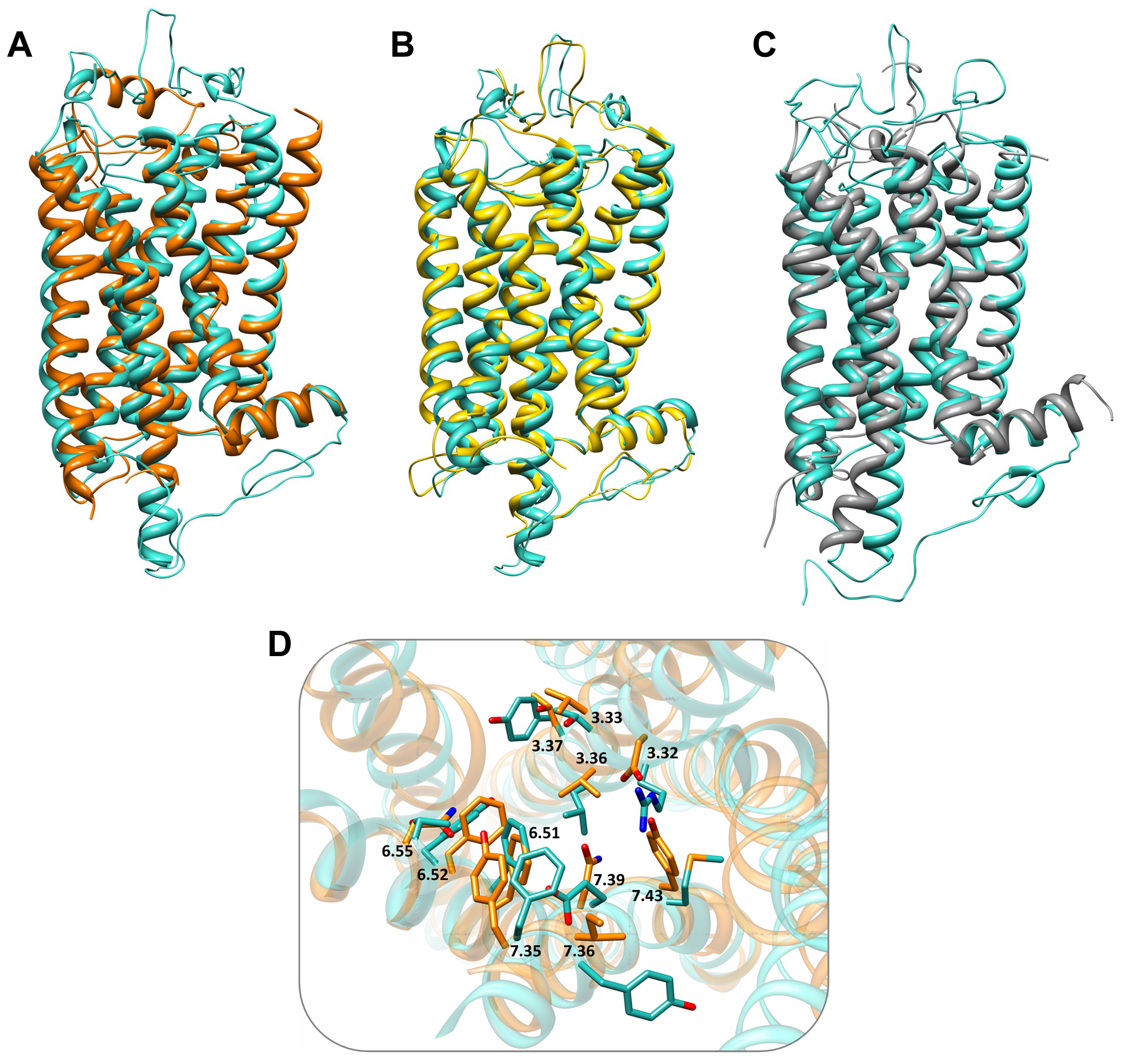

Supplement: Figure S2 — Structural superposition of the PKR1 model and GPCR X-ray templates used for homology modeling. All structures are shown in ribbon representation. PKR1 is in turquoise, human β2-adrenergic is in orange (A), bovine rhodopsin is in gold (B) and human A2A-adenosine receptor is in gray (C). (D) Superposition of the hPKR1 model and the β2-adrenergic receptor structure with emphasis on the TM-bundle binding site. The structures are shown in a view looking down on the plane of the membrane from the extracellular surface. Binding site residues experimentally known to be important for ligand binding are denoted as sticks and are labeled with Ballesteros-Weinstein numbering. The T4 lysozyme fusion protein was removed from the β2-adrenergic and the A2A-adenosine receptor structures, for clarity. Structural superposition was performed using the Matchmaker module in UCFS Chimera version 1.4.1. (TIF) [file pone.0027990.s002.tif]

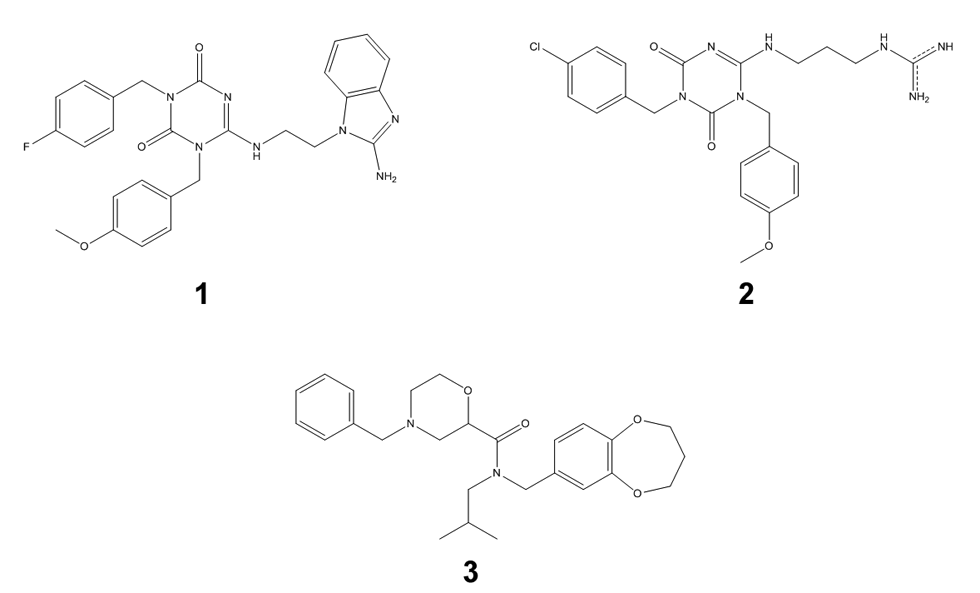

Supplement: Figure S3 — Structures of the three known PKR antagonists that were used as reference compounds for constructing ligand-based pharmacophore models. (TIF) [file pone.0027990.s003.tif]

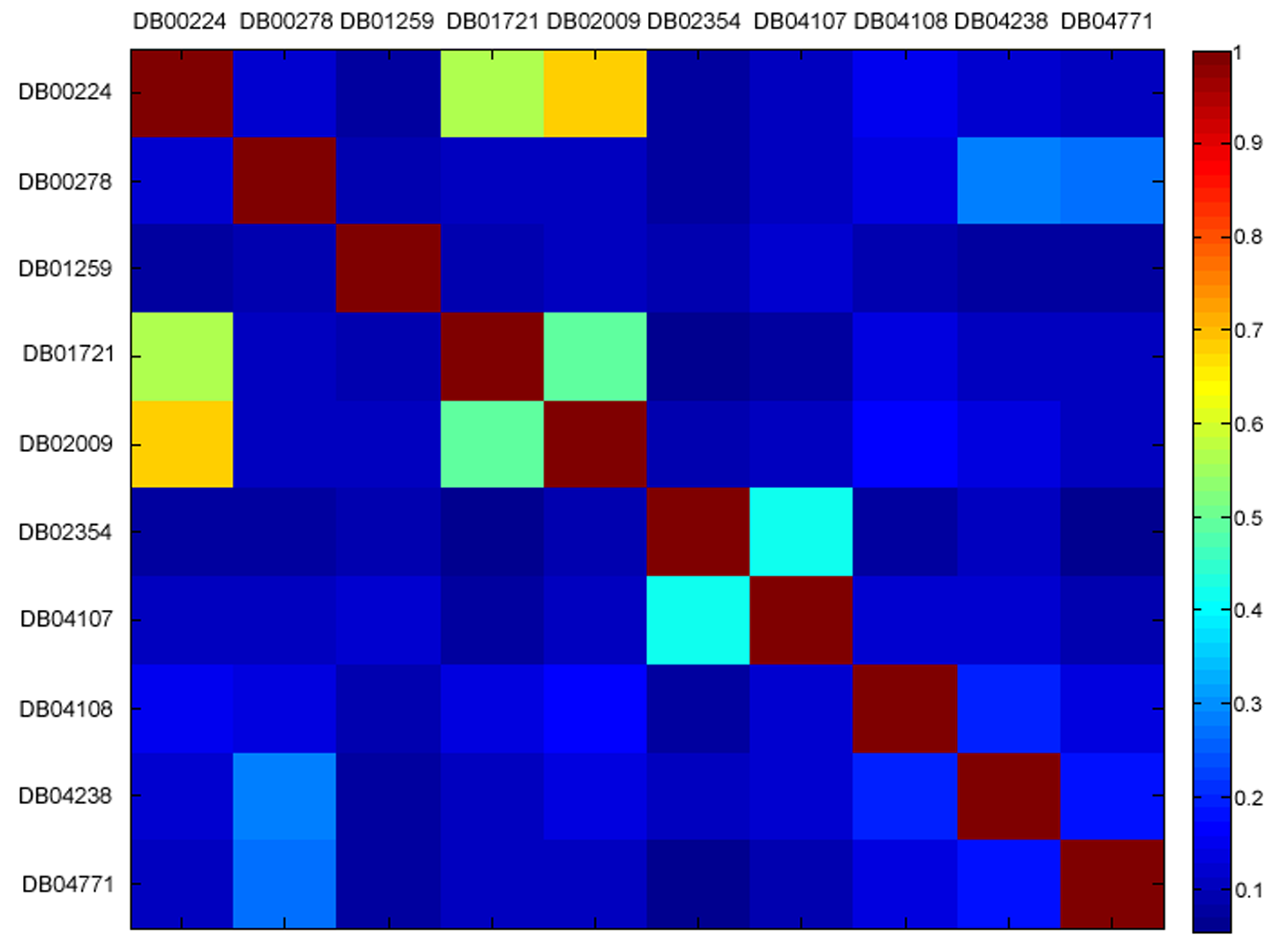

Supplement: Figure S4 — Structural similarity between the identified VLS hits plotted as a heatmap. The degree of similarity was calculated using the Tanimoto coefficient, as described in Methods, and ranges between 0 (completely dissimilar compounds) and 1 (identical compounds). Compounds with similarity values >0.85 are usually considered structurally similar. Color intensity corresponds to the similarity value according to the legend. The heatmap was prepared using Matlab version 7.10.0.499 (R2010a). (TIF) [file pone.0027990.s004.tif]

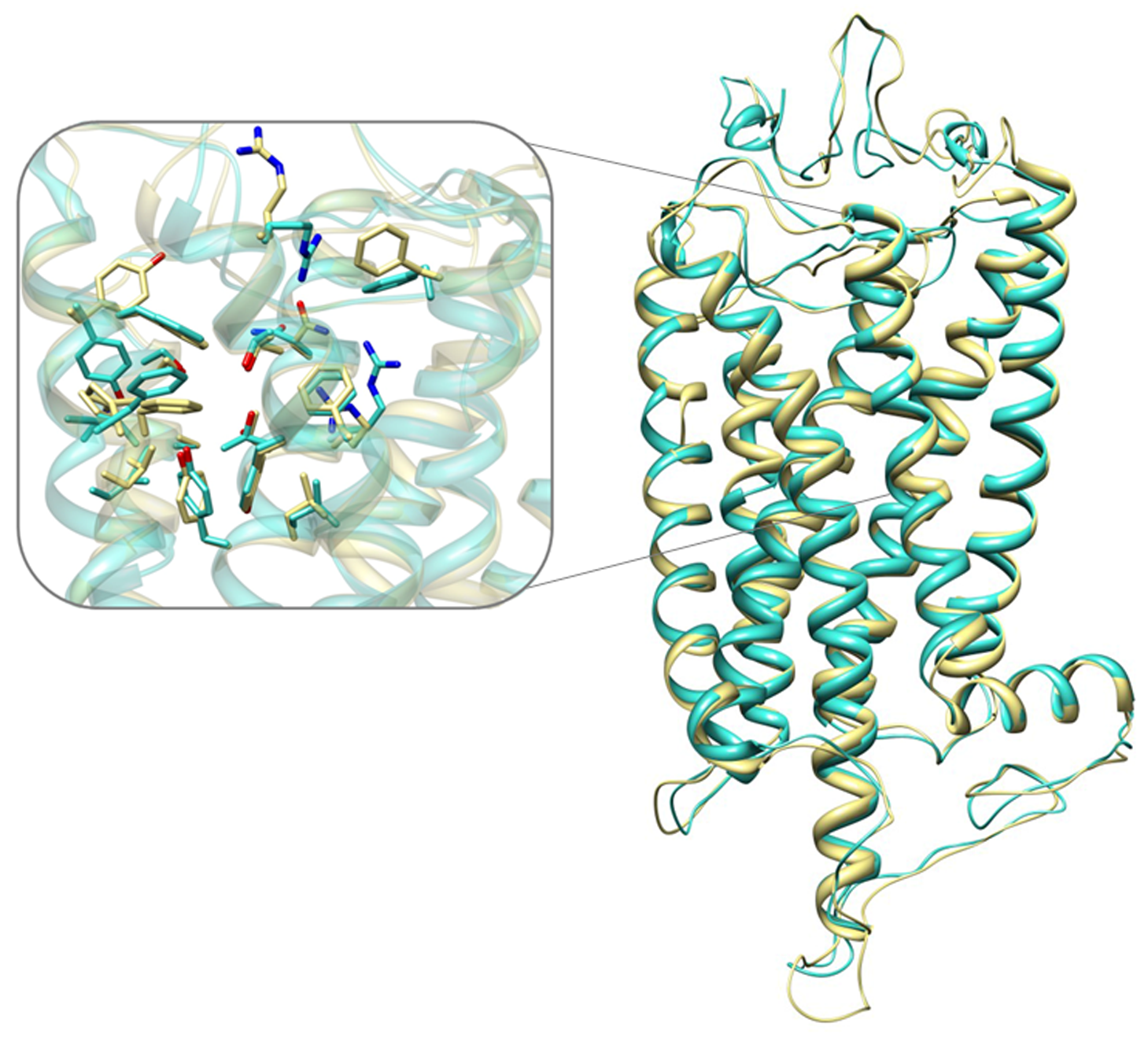

Supplement: Figure S5 — Structural superposition of human PKR1 and PKR2 models. Both structures are shown in ribbon representation, with hPKR1 in turquoise and hPKR2 in khaki. The insert shows a detailed view of the predicted transmembrane binding site, with side chains denoted as sticks. Structural superposition was performed using the Matchmaker module in UCFS Chimera version 1.4.1. (TIF) [file pone.0027990.s005.tif]

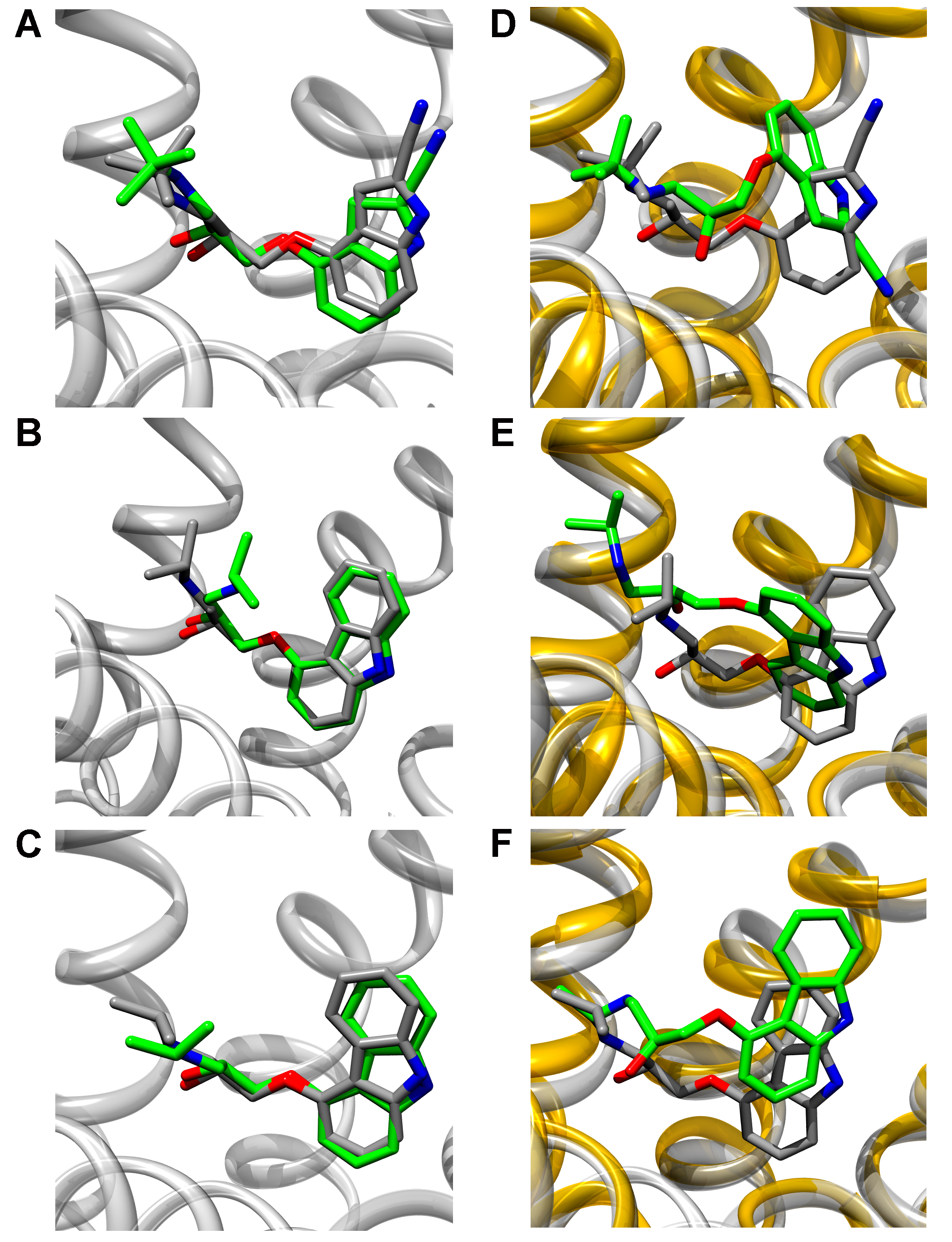

Supplement: Figure S6 — Predicted binding modes of cognate ligands redocked into crystal structures and homology models. (A) Cyanopindolol redocked to β1adr crystal structure (PDB code: 2VT4), (B) Carazolol redocked to β1adr crystal structure (2YCW), (C) Carazolol redocked to β2adr crystal structure (2RH1), (D) Cyanopindolol docked to β1adr homology model, (E) Carazolol docked to β1adr homology model and (F) Carazolol docked to β2adr homology model. The docked ligands are shown as green sticks. X-ray structures are represented as gray ribbons and the crystallized ligand is shown as gray sticks. In panels (D–F) the homology models are shown as gold ribbons. (TIF) [file pone.0027990.s006.tif]

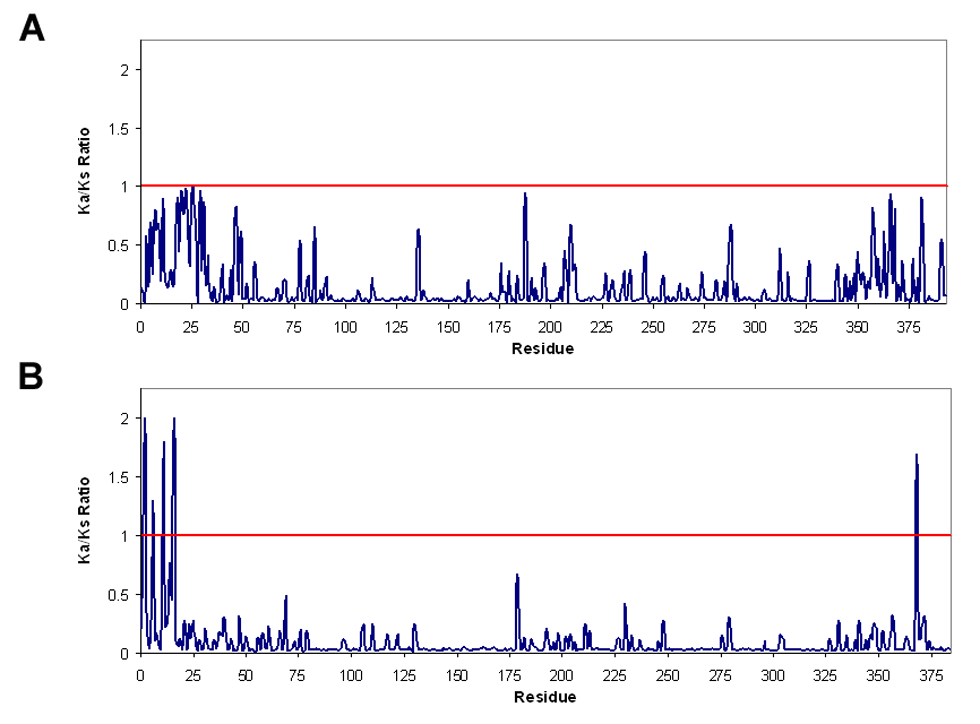

Supplement: Figure S7 — Measure of Ka/Ks ratio on the amino acid sequence of the PKR subtypes suggests positive selection acting only on PKR2. Ka/Ks ratio (ω) representing the ratio of non-synonymous (Ka) to synonymous (Ks) nucleotide substitution rates was calculated for each site for the PKR subtypes. The ratio is plotted against the amino acid position for hPKR1 (A) and hPKR2 (B). Residues showing ω>1 are indicative of positive Darwinian selection, while residues showing ω<1 are indicative of purifying selection; the ratio for neutral selection is one (indicated on the graph by a red line). Significant positive selection (p = 0.001) was detected only for PKR2, by the likelihood ratio test, and is concentrated in the N-terminus and C-terminus domains. (TIF) [file pone.0027990.s007.tif]
