# Supplementary material for: Modeling of Human Prokineticin Receptors: Interactions with Novel Small-Molecule Binders and Potential Off-Target Drugs
Source: PLoS One. 2011 Nov 21;6(11):e27990. doi: 10.1371/journal.pone.0027990 (PMC3221691; doi:10.1371/journal.pone.0027990)
Supplement: Table S1 — Potential hits identified from the ZINC database. (DOC) [file pone.0027990.s008.doc]

**Supplementary Table 1:** Potential hits identified from the ZINC database

| **ZINC ID** | **FitValue** | **Similarity** |
| --- | --- | --- |
| ZINC00628179 | 3.06605 | 0.165414 |
| ZINC01445907 | 2.94575 | 0.106195 |
| ZINC02315050 | 2.87225 | 0.121429 |
| ZINC02926931 | 3.23586 | 0.178862 |
| ZINC03056872 | 3.4984 | 0.161765 |
| ZINC03056897 | 3.54809 | 0.157895 |
| ZINC03585694 | 3.41255 | 0.161765 |
| ZINC06078193 | 2.97959 | 0.166667 |
| ZINC06078199 | 2.8914 | 0.130435 |
| ZINC06078213 | 2.93961 | 0.119718 |
| ZINC06078220 | 2.99666 | 0.142857 |
| ZINC06192971 | 2.95665 | 0.138686 |
| ZINC06192993 | 3.06201 | 0.153285 |
| ZINC06192995 | 3.01103 | 0.120567 |
| ZINC06193009 | 3.08934 | 0.152174 |
| ZINC06193011 | 3.10136 | 0.119718 |
| ZINC06193109 | 3.08048 | 0.148936 |
| ZINC06193114 | 3.03807 | 0.15 |
| ZINC06193129 | 2.92327 | 0.162791 |
| ZINC06193152 | 3.17818 | 0.135714 |
| ZINC06193179 | 3.18094 | 0.241071 |
| ZINC06193180 | 3.03722 | 0.241071 |
| ZINC06193189 | 3.06825 | 0.232143 |
| ZINC06193190 | 3.33078 | 0.232143 |
| ZINC06193192 | 3.26043 | 0.245283 |
| ZINC06193194 | 3.25129 | 0.167883 |
| ZINC06193196 | 3.33153 | 0.17037 |
| ZINC06193197 | 3.25546 | 0.17037 |
| ZINC06193199 | 3.0235 | 0.179104 |
| ZINC06193200 | 3.24043 | 0.203008 |
| ZINC06193202 | 3.04896 | 0.182482 |
| ZINC06193205 | 3.08058 | 0.171642 |
| ZINC06193206 | 3.30711 | 0.169118 |
| ZINC06193207 | 3.15706 | 0.194915 |
| ZINC06193218 | 3.17755 | 0.175573 |
| ZINC06193220 | 3.36977 | 0.175573 |
| ZINC06193238 | 3.51481 | 0.136691 |
| ZINC06193246 | 3.3722 | 0.156716 |
| ZINC06193252 | 3.20001 | 0.136691 |
| ZINC06193253 | 3.19198 | 0.136691 |
| ZINC06193261 | 3.15298 | 0.169118 |
| ZINC06193308 | 2.94685 | 0.146154 |
| ZINC06193311 | 2.98769 | 0.176923 |
| ZINC06193317 | 2.93659 | 0.162791 |
| ZINC06194908 | 3.32183 | 0.122302 |
| ZINC06195344 | 2.92856 | 0.115646 |
| ZINC06197335 | 3.07273 | 0.129771 |
| ZINC06197341 | 3.06722 | 0.151515 |
| **ZINC ID** | **FitValue** | **Similarity** |
| ZINC06197610 | 3.11081 | 0.165414 |
| ZINC06198478 | 3.28386 | 0.173913 |
| ZINC06198533 | 3.71365 | 0.247619 |
| ZINC06198535 | 2.91746 | 0.238532 |
| ZINC06198537 | 3.14825 | 0.236364 |
| ZINC06198539 | 3.27184 | 0.191304 |
| ZINC06198540 | 3.34598 | 0.2 |
| ZINC06198543 | 3.72167 | 0.210526 |
| ZINC06198560 | 2.99031 | 0.168142 |
| ZINC06198574 | 3.22183 | 0.152542 |
| ZINC06198583 | 3.01505 | 0.178571 |
| ZINC06239787 | 3.20385 | 0.238532 |
| ZINC06283101 | 3.00218 | 0.137681 |
| ZINC06283194 | 3.10007 | 0.140741 |
| ZINC06283196 | 2.97553 | 0.139706 |
| ZINC06283206 | 3.07458 | 0.142857 |
| ZINC06283210 | 3.00218 | 0.137681 |
| ZINC06444209 | 2.86523 | 0.116279 |
| ZINC06445072 | 2.8959 | 0.135714 |
| ZINC06445495 | 3.06093 | 0.230769 |
| ZINC06445849 | 2.97601 | 0.131944 |
| ZINC06445858 | 2.95647 | 0.131034 |
| ZINC06445927 | 2.95141 | 0.131944 |
| ZINC06445931 | 3.13397 | 0.131034 |
| ZINC06498723 | 3.13449 | 0.073826 |
| ZINC08636807 | 3.236 | 0.086331 |
| ZINC08685681 | 3.4399 | 0.106383 |
| ZINC08778701 | 3.27745 | 0.115108 |
| ZINC08778797 | 3.22141 | 0.150376 |
| ZINC08779808 | 3.07174 | 0.085938 |
| ZINC08814222 | 3.00751 | 0.164286 |
| ZINC08814227 | 3.45315 | 0.157895 |
| ZINC08814228 | 3.54131 | 0.160305 |
| ZINC08814239 | 2.98058 | 0.161972 |
| ZINC08814244 | 3.39094 | 0.207407 |
| ZINC08814256 | 3.24196 | 0.152672 |
| ZINC08814294 | 3.34854 | 0.096552 |
| ZINC08835084 | 3.28155 | 0.066116 |
| ZINC08837536 | 3.13094 | 0.0625 |
| ZINC08856752 | 2.98241 | 0.142857 |
| ZINC08856754 | 3.1731 | 0.142857 |
| ZINC08857514 | 3.1372 | 0.10219 |
| ZINC08904955 | 3.02755 | 0.108108 |
| ZINC08926714 | 2.97948 | 0.111111 |
| ZINC09008327 | 3.29033 | 0.137931 |
| ZINC09019221 | 3.27519 | 0.125 |
| ZINC09019750 | 3.10487 | 0.114035 |
| ZINC09019892 | 3.20577 | 0.127119 |
| ZINC09019908 | 2.89045 | 0.125 |
| ZINC09019917 | 3.19037 | 0.105691 |
| **ZINC ID** | **FitValue** | **Similarity** |
| ZINC09020093 | 3.21846 | 0.112069 |
| ZINC09046727 | 3.1579 | 0.07438 |
| ZINC09123966 | 3.04293 | 0.12605 |
| ZINC09123967 | 3.27071 | 0.12605 |
| ZINC09191506 | 3.07483 | 0.117647 |
| ZINC09281321 | 2.87829 | 0.147826 |
| ZINC09282483 | 2.89958 | 0.117117 |
| ZINC09312960 | 3.28983 | 0.107438 |
| ZINC09313125 | 3.00438 | 0.122807 |
| ZINC09314958 | 3.16174 | 0.134752 |
| ZINC09334053 | 3.01278 | 0.106557 |
| ZINC09353286 | 2.93014 | 0.117647 |
| ZINC09354212 | 3.04535 | 0.144068 |
| ZINC09354280 | 2.89845 | 0.088 |
| ZINC09354284 | 3.13147 | 0.088 |
| ZINC09358546 | 2.8893 | 0.1875 |
| ZINC09442268 | 2.97688 | 0.165354 |
| ZINC09460957 | 3.05209 | 0.130435 |
| ZINC09460970 | 3.04494 | 0.127119 |
| ZINC09460971 | 2.95787 | 0.127119 |
| ZINC09463889 | 3.08832 | 0.121951 |
| ZINC09465882 | 3.04797 | 0.09375 |
| ZINC09514986 | 2.91854 | 0.159292 |
| ZINC09648225 | 3.38062 | 0.07971 |
| ZINC09648260 | 3.25742 | 0.072993 |
| ZINC09648314 | 3.18681 | 0.109489 |
| ZINC09648315 | 3.09193 | 0.107914 |
| ZINC09648370 | 3.46958 | 0.116788 |
| ZINC09648400 | 3.17355 | 0.090909 |
| ZINC09680548 | 3.11354 | 0.147059 |
| ZINC09680553 | 3.46607 | 0.149254 |
| ZINC09680555 | 3.10239 | 0.150376 |
| ZINC09716022 | 3.11355 | 0.104762 |
| ZINC10063475 | 2.86005 | 0.141791 |
| ZINC10063592 | 2.86584 | 0.175 |
| ZINC10063594 | 3.31422 | 0.184615 |
| ZINC10063601 | 2.86112 | 0.143939 |
| ZINC10063607 | 3.04998 | 0.135338 |
| ZINC10063611 | 3.46681 | 0.107143 |
| ZINC10063613 | 3.14877 | 0.111111 |
| ZINC10063618 | 3.00834 | 0.140741 |
| ZINC10063623 | 3.2357 | 0.163934 |
| ZINC10063626 | 3.24957 | 0.174242 |
| ZINC10063637 | 3.10772 | 0.19469 |
| ZINC10063639 | 3.43761 | 0.160584 |
| ZINC10063641 | 2.97163 | 0.161765 |
| ZINC10063648 | 3.09252 | 0.2 |
| ZINC10063663 | 3.18418 | 0.181818 |
| ZINC10063676 | 3.33791 | 0.167939 |
| ZINC10063681 | 3.06086 | 0.178295 |
| **ZINC ID** | **FitValue** | **Similarity** |
| ZINC10231700 | 3.08108 | 0.101695 |
| ZINC10231701 | 2.97165 | 0.140351 |
| ZINC10231702 | 2.91356 | 0.166667 |
| ZINC10231703 | 3.06727 | 0.166667 |
| ZINC10231795 | 2.91412 | 0.142857 |
| ZINC10386288 | 3.33939 | 0.15942 |
| ZINC10510640 | 3.09356 | 0.105263 |
| ZINC11392822 | 2.96858 | 0.142857 |
| ZINC12100575 | 3.03251 | 0.110169 |
| ZINC12535966 | 2.9011 | 0.162162 |
| ZINC13752130 | 3.38464 | 0.109244 |
| ZINC13752132 | 3.06641 | 0.109244 |
| ZINC14156069 | 2.96069 | 0.176923 |
| ZINC14156224 | 3.4201 | 0.165414 |
| ZINC14156502 | 3.2228 | 0.107692 |
| ZINC14156642 | 3.03583 | 0.130769 |
| ZINC14156785 | 3.20963 | 0.179688 |
| ZINC14156858 | 3.48784 | 0.159091 |
| ZINC14156934 | 3.04764 | 0.179688 |
| ZINC14157018 | 3.25647 | 0.128788 |
| ZINC14157166 | 3.27685 | 0.176923 |
| ZINC14157277 | 2.99764 | 0.104478 |
| ZINC14157389 | 3.13442 | 0.104478 |
| ZINC14158164 | 3.40911 | 0.108108 |
| ZINC14158312 | 2.95811 | 0.136364 |
| ZINC14158388 | 3.00569 | 0.121212 |
| ZINC14158835 | 3.50139 | 0.135135 |
| ZINC14159080 | 3.10045 | 0.102941 |
| ZINC14160357 | 3.2764 | 0.087591 |
| ZINC14161365 | 3.2351 | 0.174242 |
| ZINC14290632 | 2.98026 | 0.162963 |
| ZINC14293882 | 2.95422 | 0.114286 |
| ZINC14806775 | 3.14306 | 0.171429 |
| ZINC15155807 | 3.36692 | 0.161765 |
| ZINC16044681 | 3.04493 | 0.151786 |
| ZINC16645801 | 3.0376 | 0.109589 |
| ZINC16662134 | 2.91337 | 0.106557 |
| ZINC16958124 | 3.04931 | 0.090164 |
| ZINC17147578 | 2.89466 | 0.09322 |
| ZINC17215248 | 3.03702 | 0.097902 |
| ZINC17215258 | 3.09253 | 0.092437 |
| ZINC17215277 | 3.15008 | 0.111111 |
| ZINC18077315 | 3.34967 | 0.136054 |
| ZINC18077318 | 3.40371 | 0.136054 |
| ZINC18077321 | 3.52186 | 0.136054 |
| ZINC18125045 | 2.93151 | 0.122449 |
| ZINC19294395 | 3.44636 | 0.157895 |
| ZINC20075570 | 2.95919 | 0.129496 |
| ZINC20151314 | 2.99541 | 0.087591 |
| ZINC20151321 | 3.04159 | 0.123188 |
| **ZINC ID** | **FitValue** | **Similarity** |
| ZINC20151325 | 2.97341 | 0.141791 |
| ZINC20453694 | 2.85815 | 0.153846 |
| ZINC20590768 | 2.91772 | 0.107914 |
| ZINC20737568 | 2.86208 | 0.096552 |
| ZINC21698836 | 2.93141 | 0.069444 |
| ZINC22276774 | 2.90756 | 0.087719 |
| ZINC22277159 | 3.17097 | 0.09009 |
| ZINC22926802 | 2.97877 | 0.148438 |
| ZINC23131651 | 3.22356 | 0.262626 |
| ZINC23975472 | 3.04016 | 0.134454 |
| ZINC25402306 | 3.03406 | 0.144928 |
| ZINC33025277 | 3.34441 | 0.089655 |
