# Supplementary material for: Modeling of Human Prokineticin Receptors: Interactions with Novel Small-Molecule Binders and Potential Off-Target Drugs
Source: PLoS One. 2011 Nov 21;6(11):e27990. doi: 10.1371/journal.pone.0027990 (PMC3221691; doi:10.1371/journal.pone.0027990)
Supplement: Table S2 — Ligand RMSD values and contact analysis for cognate ligand docking to β1adr and β2adr crystal structures and homology models. (DOC) [file pone.0027990.s009.doc]

**Supplementary table 2:** Ligand RMSD values and contact analysis for cognate ligand docking to β1adr and β2adr crystal structures and homology models

| Receptor | Ligand | Ligand RMSD (Å) | Atomic contacts/ Reference | Correct contact strength  (% reference) | Correct interacting residues/ Reference ^a^ | Correct residue strength  (% reference) | Critical contacts ^b^ |
| --- | --- | --- | --- | --- | --- | --- | --- |
| β1adr Xray | Cyanopindolol | 0.8972 | 47 / 68 | 69.11 | 12 / 15 | 80 | D121, N310, N329 |
| β1adr Xray | Carazolol | 1.2712 | 46 / 68 | 67.64 | 11 / 14 | 78.57 | D121, N329 |
| β2adr Xray | Carazolol | 0.89294 | 53 / 75 | 70.66 | 15 / 17 | 88.23 | D113, N312 |
| β1adr model | Cyanopindolol | 3.4811 | 15 / 68 | 22.05 | 10 / 15 | 66.66 | D121, S211, S212, S215, F306 |
| β1adr model | Carazolol | 4.0705 | 15 / 68 | 22.05 | 9 / 15 | 60 | D121, Y207, N329 |
| β2adr model | Carazolol | 2.9893 | 19 / 75 | 25.33 | 8 / 17 | 47.05 | D113, S203 |

^a^ residue involved in heavy atom contact.

^b^ critical contact-forming residues in Xray are underlined.
